# ARF-like GTPase 8B orchestrates lipophagy and exocytosis to drive single-stranded RNA virus replication

**DOI:** 10.1186/s12967-026-08417-2

**Published:** 2026-06-13

**Authors:** Bonan Lv, Xingran Wang, Ying Zhou, Zihan Su, Yidan Sun, Li Zhou, Yang Lu, Zishu Pan, Xiao-Feng Tang, Chao Shen

**Affiliations:** 1 State Key Laboratory of Virology and Biosafety, College of Life Sciences, Wuhan University, Wuhan, 430072 China; 2 Hubei Key Laboratory of Cell Homeostasis, College of Life Sciences, Wuhan University, Wuhan, 430072 China; 3 ABSL-III Laboratory at Center for Animal Experiment, Wuhan University School of Medicine, Wuhan, 430071 China

**Keywords:** Foot-and-mouth disease virus, ARL8B, LD degradation, Lysosomal–exosome pathways, Small-molecule inhibitors

## Abstract

**Background:**

Single-stranded RNA (ssRNA) viruses, including foot‑and‑mouth disease virus (FMDV), enterovirus 71 (EV71), and vesicular stomatitis virus (VSV), reprogram host lipid metabolism to facilitate replication. However, the mechanisms governing virus‑induced lipid droplet (LD) degradation remain poorly defined, which limits the development of host‑directed antiviral strategies.

**Methods:**

We employed virological, biochemical, and imaging approaches to investigate the role of the lysosomal GTPase ARL8B in ssRNA virus infection. Lipid droplet dynamics, lipophagy, and lysosomal exocytosis were assessed using confocal microscopy and biochemical assays. Small‑molecule inhibitors targeting ARL8B were identified through virtual screening and validated in vitro for antiviral activity. The in vivo efficacy of selected inhibitors was evaluated in a murine EV71 infection model, with disease severity and survival as endpoints.

**Results:**

ARL8B was identified as a central host factor that promotes replication of multiple ssRNA viruses by coordinating LD degradation with viral egress. Mechanistically, ARL8B drove lysosome‑dependent LD breakdown via selective lipophagy, releasing free fatty acids that fuel membrane remodeling within viral replication complexes. Concurrently, ARL8B facilitated viral exit through a noncanonical lysosomal–exosome pathway, enhancing progeny virus dissemination. Virtual screening yielded small‑molecule ARL8B inhibitors that potently suppressed ssRNA virus replication in vitro. In a murine EV71 infection model, these inhibitors provided robust protection and substantially reduced disease severity.

**Conclusions:**

Our findings establish ARL8B as a key host factor coupling lipid catabolism with viral export, and unveil a class of broad‑spectrum antiviral candidates targeting a lipid reprogramming node essential for multiple pathogenic RNA viruses. This study provides a mechanistic framework for developing host‑directed antiviral therapies against ssRNA viruses.

**Supplementary Information:**

The online version contains supplementary material available at 10.1186/s12967-026-08417-2.

## Introduction

Lipids are essential organic biomolecules that provide structural integrity for cellular membranes, participate in signaling cascades, and serve as energy reservoirs [1]. Among lipid-related organelles, lipid droplets (LDs) are neutral lipid storage compartments that act as central hubs of lipid homeostasis and have been increasingly recognized as critical players in the life cycles of many viruses [2, 3]. LDs are not inert depots; they undergo dynamic turnover through a selective autophagic process termed lipophagy, which integrates LD catabolism into the lysosome–autophagy pathway. Three autophagic routes contribute to triglyceride degradation: macroautophagy, microautophagy, and chaperone‑mediated autophagy [4]. In macrolipophagy, LDs are sequestered by autophagosomes to form lipophagosomes, which subsequently fuse with lysosomes to enable LD degradation. Microlipophagy does not require full enclosure of LDs; instead, lysosomes transiently dock with LDs, allowing direct lipid transfer. Chaperone‑mediated autophagy is partly regulated by adipose triglyceride lipase (ATGL): autophagic degradation of perilipin proteins permits ATGL to access the LD surface, stimulating LD breakdown. Heat shock protein 70 (HSP70) directly interacts with perilipin 2 and perilipin 3 on LDs by recognizing a specific pentapeptide motif [5, 6]; HSP70 then transports these proteins to lysosomes, where uptake and degradation are mediated by lysosome‑associated membrane protein 2. LD catabolism and lipophagy function as complementary pathways: classical LD degradation digests large LDs into smaller droplets, and lipophagy degrades the smaller ones. ATGL inhibition results in accumulation of large LDs, whereas lysosomal acid lipase deficiency leads to small LD accumulation [7]. Importantly, impairment of lipid autophagy sensitizes cells to death stimuli and may contribute to the pathogenesis of nonalcoholic fatty liver disease and metabolic syndrome [8]. Despite this knowledge, the regulatory relationship between intracellular lipids and autophagy remains incompletely understood, and how these processes contribute to viral life cycles is largely unclear.

Numerous positive‑sense single‑stranded RNA viruses, including human and animal pathogens with major clinical and economic impacts, exploit host lipid metabolism to support their replication and assembly. Foot‑and‑mouth disease virus (FMDV), a member of the genus Aphthovirus within the family Picornaviridae, causes a highly contagious and devastating disease in even‑toed ungulates, characterized by vesicular lesions in the mouth, feet, and mammary glands, accompanied by fever, salivation, and lameness [9–11]. Owing to its rapid transmissibility, FMDV poses a persistent threat to livestock industries worldwide [12]. Enterovirus 71 (EV71), another Picornaviridae family member [13], exhibits high neurotropism and can cause severe manifestations such as encephalitis, poliomyelitis‑like paralysis, and even death. The mechanisms underlying EV71 pathogenesis remain elusive, and no effective vaccines or antiviral drugs are currently available [14]. Negative‑strand RNA viruses also remodel host lipid species during infection; for example, vesicular stomatitis virus (VSV), a member of the Rhabdoviridae family, substantially alters the host lipidome [15]. Investigations into the roles of host lipids in viral infection are therefore essential for understanding pathogenesis and for advancing the development of vaccines and antiviral therapeutics.

Viral infection frequently induces the formation of autophagosomes, which subsequently fuse with lysosomes to form autolysosomes [16]. The acidic environment of autolysosomes activates enzymes that degrade aggregation‑prone or misfolded proteins, dysfunctional or excess organelles, and invading pathogens [17]. Autophagy may positively regulate the replication of picornaviruses, but the underlying molecular details remain incompletely defined [18, 19].

Exosomes are extracellular vesicles (EVs) of 30–150 nm in diameter that originate from multivesicular bodies (MVBs), the plasma membrane (microvesicles), or other organelles such as autophagosomes [20, 21]. Both RNA and DNA viruses can hijack the endosomal sorting complex required for transport (ESCRT) machinery and Rab GTPases to acquire envelopes or to facilitate non‑lytic release. For instance, dengue virus obtains its envelope within MVBs [22], and EVs promote the export of diverse viruses, including hepatitis A virus [20, 21], hepatitis C virus, and dengue virus [23]. Enveloped and non‑enveloped viruses alike utilize EVs pathways to secrete viral particles, proteins, and nucleic acids, thereby enhancing infection by transferring viral components to recipient cells and modulating host responses [24].

Adenosine diphosphate‑ribosylation factor‑like GTPase 8B (ARL8B) is a small (22‑kDa) GTPase that, in its GTP‑bound state, localizes to lysosomes and serves as a master regulator of their bidirectional transport along microtubules [25, 26]. Beyond its canonical role in lysosomal positioning, ARL8B directs membrane trafficking by recruiting the homotypic fusion and protein sorting (HOPS) complex, thereby facilitating late endosome‑lysosome fusion and antigen delivery [27]. Its functional repertoire extends to host‑pathogen interactions: during coronavirus infection, ARL8B mediates a non‑lytic lysosomal exocytosis pathway that co‑releases viral particles and the chaperone BiP [28]; it has also been implicated in modulating host lipid turnover [29, 30] and autophagic flux [31]. Collectively, these diverse functions position ARL8B as a potential nexus linking the autophagy‑lysosome axis to lipid metabolism.

However, the role of lipid metabolism and LD dynamics in cells infected with picornaviruses such as FMDV remains poorly understood. We previously reported that FMDV hijacks host lipid metabolism to promote its replication, underscoring the critical involvement of LDs in the viral life cycle [32, 33]. Despite this, the molecular mechanisms by which FMDV commandeers the lysosomal machinery to reprogram lipid homeostasis, and the potential interplay with extracellular vesicles (EVs) biogenesis, have not been explored. In this study, we elucidate a previously unrecognized reciprocal regulation centered on ARL8B that governs LD degradation via lipophagy and coordinates EVs biogenesis during FMDV infection. Our findings reveal a novel virus–host interplay that sustains a pro‑viral cellular environment and provide a theoretical framework for developing antiviral strategies against foot‑and‑mouth disease.

## Materials and methods

### Cell lines and virus

Foot-and-mouth disease virus type O (Akesu/58/2002) was provided by the Lanzhou Institute of Veterinary Medicine, Chinese Academy of Agricultural Sciences. Virus titers were measured using a plaque assay. The BHK-21 (baby hamster kidney), PK-15 (pig kidney), and Vero (African green monkey kidney) cell lines were obtained from the China Center for Type Culture Collection (CCTCC). Their respective accession numbers and Research Resource Identifiers (RRIDs) are: BHK-21 (CCTCC No. GDC0010; RRID: CVCL_1914), PK-15 (CCTCC No. GDC0061; RRID: CVCL_2160), and Vero (CCTCC No. GDC0029; RRID: CVCL_0059). Poliovirus (GDV057), enterovirus 71 (GDV067), and vesicular stomatitis virus (GDV027) were gifts from the CCTCC.

### Cell culture

BHK-21 cells were grown in T25 flasks with 4–6 mL of minimum essential medium (MEM; NEST Biotechnology) containing 10% fetal bovine serum (Every Green, Zhejiang Tianhang Biotechnology) and 1% penicillin–streptomycin. All cell lines were maintained at 37 °C in a humidified 5% CO₂ incubator. Cells were revived and cultured according to CCTCC protocols. Authentication via STR profiling and absence of bacterial, fungal, and mycoplasma contamination were confirmed by CCTCC quality reports. In addition, cells were routinely screened for mycoplasma using PCR during the study.

### Plasmid construction and transfection

Total RNA extracted from BHK-21 cells was reverse transcribed into cDNA. Target gene fragments were amplified with Phanta Max Super-Fidelity DNA Polymerase (Vazyme) and cloned into the pHAGE-CMV-MCS-IZsGreen vector (Thermo Fisher) using the ClonExpress II One Step Cloning Kit (Vazyme). Plasmids were verified by restriction digestion and sequencing.

For ARL8B knockdown, a 21-nt oligonucleotide was designed, annealed to form siRNA duplexes, and inserted between the HindIII and BglII sites of the pSUPER.retro.puro vector (Oligoengine). Three siRNA sequences were used: Si-ARL8B#1: GACCACUUUCGUCAAUGUCAUDTDT; Si-ARL8B#2: UUGCAGAGGAGUCAAUGCUAUDTDT; Si-ARL8B#3: CUCUCGAAAUGAGCUCCAUAADTDT. Correct insertion was confirmed by EcoRI and HindIII digestion.

For transfection, cells seeded in 12-well plates (~ 60% confluency) were treated with a mixture of Lipofectamine 2000 (Thermo Fisher) and plasmid DNA in Opti-MEM (Thermo Fisher). Medium was refreshed 4–6 h post-transfection.

### Generation of knockout cell lines by electroporation

sgRNAs targeting exons 4 and 6 of ARL8B were designed using the CRISPR design tool (http://crispr.mit.edu/), following the “20 N + NGG” rule. Sense strands were modified with a 5′ CACC overhang. Annealed sgRNAs were ligated into a CRISPR vector using T_4_ DNA ligase (Thermo Fisher) and transformed into Trans1-T1 competent cells. Correct clones were identified by PCR.

For electroporation, 3 × 10⁶ cells were resuspended in 600 µL PBS, mixed with 30 µg of endotoxin-free CRISPR plasmid, and transferred to a 100-µL electroporation tip. Electroporation was performed at 200 V for 20 ms using a Thermo Fisher electroporator. Cells were then plated in pre-warmed medium in 6-well plates. Monoclonal lines were screened by PCR and western blotting; positive clones were expanded for subsequent experiments.

### RNA extraction and RT-qPCR

Total RNA was isolated from cells in 12-well plates using 500 µL RNAiso Plus (Takara) with incubation for 30 min at 4 °C. Chloroform extraction was followed by isopropanol precipitation. First-strand cDNA was generated with the Hifair II 1st Strand cDNA Synthesis Kit (Yeasen). Quantitative PCR was carried out on a CFX96 system (Bio-Rad) with SYBR Green chemistry. GAPDH was used as the endogenous reference, and the 2^−ΔΔ^Ct method was applied to calculate relative expression levels. All primer sequences are listed in Table 1.

**Table 1:** Primers used in the research

Genes	Species	Sequences
*GAPDH* *FMDV* *ARL8B*	*Golden hamster* *Golden hamster* *Golden hamster*	F: AAGGCCATCACCATCTTCCA R: GCCAGTAGACTCCACAACATAC F: GAACACATTCTTTACACCAGGAT R: CATATCTTTGCCAATCAACATCAG F: AATGTCATCGCGTCAGGTCA R: AACCGGGGTTGTCCTCCTAT
*EV71*	*Golden hamster*	F: GCTCTATAGGAGATAGTGTGAGTAGGG
		R: ATGACTGCTCACCTGCGTGTT
*PV*	*Golden hamster*	F: TCCGGCCCCTGAATGCGGCT
		R: TGTCACCATAAGCAGCC
*VSV*	*Golden hamster*	F: TAATTCCACGAAGCACCGAG
		R: AATGAGCAACAGTCAAGGCA

### Western blotting

Following PBS washes, cells were lysed on ice with RIPA buffer. After centrifugation, the supernatant was combined with 2× Laemmli buffer, heated at 95 °C for 5 min, and recentrifuged. Proteins were resolved on 10% or 15% SDS-PAGE gels and transferred onto PVDF membranes. Membranes were blocked with 5% skim milk (1–2 h) and then probed with primary antibodies overnight at 4 °C. After washing, HRP-conjugated secondary antibodies were applied for 2 h at room temperature. Protein bands were visualized by enhanced chemiluminescence. Antibody details are provided in Table 2.

**Table 2:** Antibodies used in the research

Antibody	Company	Dilution ratio
GAPDH	Huabio	1:2,000
FMDV 3D	Huabio	1:2,000
EV71 3D	Huabio	1:2,000
Myc	Huabio	1:2,000
Flag	Huabio	1:1,000
FMDV 2 C	Huabio	1:2,000
FMDV dsRNA	English and Scientific Consulting Kft	1:200
ARL8B	ABclonal	1:10,000
Goat anti-rabbit IgG	ABclonal	1:10,000
Goat anti-mouse IgG	ABclonal	1:10,000
RAB7	Huabio	1:1,000
Calnexin	Huabio	1:1,000
LAMP1	Huabio	1:1,000
LC3	ABclonal	1:1,000
BIP	ABclonal	1:1,000
P62	Huabio	1:1,000
ERP72	Huabio	1:1,000
GM130	Huabio	1:1,000
Goat anti-mouse IgG (H + L) antibody, FITC conjugate	ABclonal	1:100
Goat anti-mouse IgG (H + L) antibody, TRITC conjugate	ABclonal	1:100

### Viral titer determination

BHK-21 cells were trypsinized, seeded into 96-well plates, and infected with serial dilutions of virus stock in serum-free MEM (100 µL/well, eight replicates per dilution). Control wells received virus-free medium. MEM containing 2% FBS (100 µL) was then added to each well. Plates were incubated at 37 °C with 5% CO₂, and cytopathic effects were recorded daily over 3 days. The number of wells showing cytopathology at each dilution was counted.

### Cytotoxicity assay

Test compounds (Brefeldin A, CID-1067700, Chloroquine, HY-Q31266, and HY-W051988; MCE) were diluted in DMSO to eight concentrations. Compounds were applied to cells in 96-well plates (eight replicates per concentration). After 24 h, CCK-8 reagent (Yeasen; 10% v/v) was added, and plates were incubated for 2 h at 37 °C. Absorbance at 450 nm was measured using a microplate reader (Thermo Fisher). Cell viability was determined as (OD₄₅₀ sample − OD₄₅₀ blank) / (OD₄₅₀ solvent control − OD₄₅₀ blank).

### Co-immunoprecipitation

Cells in T25 flasks were washed with PBS and lysed with 1 mL IP Lysis Buffer (Beyotime) supplemented with protease inhibitors for 10 min at 4 °C with gentle shaking. Lysates were collected by scraping. Antibodies were pre-incubated with magnetic beads (MCE) in binding/wash buffer (1:2000 dilution) overnight at 4 °C on a rotator. Cleared lysates (400 µL) were then mixed with the antibody-bead complexes and incubated for 2 h at 4 °C. After four washes with binding/wash buffer, bound proteins were eluted under denaturing conditions for subsequent immunoblotting.

### Immunofluorescence

Cells cultured in 24-well plates (60–70% confluence) were fixed with 4% paraformaldehyde (30 min), permeabilized with 0.5% Triton X-100 in PBS (12 min), and blocked with 5% BSA (30 min). Primary antibody diluted in 1% BSA was applied overnight at 4 °C. After washing with PBS containing 0.5% Triton X-100, cells were incubated with appropriate secondary antibody for 2 h at room temperature. Images were acquired on a BC43 spinning-disk confocal microscope (Oxford Instruments Andor).

### Free fatty acid assay

Cells were treated with 200 µM oleic acid for 24 h, followed by incubation in serum-free DMEM for 4 h. Free fatty acids were measured using the Free Fatty Acid Assay Kit (ab65341, Abcam). In brief, cells were harvested in cold PBS and centrifuged at 600 × g for 10 min (4 °C). The pellet was resuspended in 200 µL chloroform containing 1% Triton X-100, homogenized, and kept on ice for 20 min. After centrifugation, the lower organic phase was collected, dried under vacuum at 50 °C, and dissolved in 200 µL Fatty Acid Assay Buffer. For quantification, 50 µL of sample or standard was mixed with 2 µL ACS Reagent and incubated at 37 °C for 30 min. Then 50 µL Reaction Mix was added, and after a further 30 min incubation at 37 °C in the dark, absorbance was read at 570 nm [34].

### Luciferase reporter assay

Cells in 96-well plates were transfected with pGLuc-Dura-SV40-C plasmid (Beyotime) and cultured for 72 h, with drug treatments applied as indicated. For supernatant assays, 50–200 µL culture medium was collected and equilibrated to room temperature. For lysate assays, cells were washed with PBS and lysed with 100 µL lysis buffer (Beyotime) per well with shaking for 15 min. A 5–20 µL aliquot of sample was transferred to an assay plate, mixed with 50 µL detection working solution, and incubated at 25 °C for 5–10 min. Luminescence was measured using a GloMax 20/20 Luminometer.

### Structured illumination microscopy (SIM)

SIM imaging was performed on a Multi-SIM system (Beijing NanoInsights-Tech) with a 100×/1.49 NA oil-immersion objective (Nikon). Lasers at 405, 488, 561, and 640 nm and a sCMOS camera (Photometrics Kinetix) were used. Samples on coverslips were imaged with immersion oil (refractive index 1.518). System resolution was calibrated with 100-nm fluorescent beads. Image stacks were reconstructed using SI-Recon 2.23.3 (NanoInsights) with a pixel size of 30.6 nm, channel-specific optical transfer functions, a Wiener filter constant of 0.01 (2D) or 0.005 (3D), and background removal. Reconstructed images were denoised via total variation constraint, and channel registration was corrected using fluorescent beads.

### Dynamic refractive index imaging of live cells

BHK-21 cells were allowed to adhere for 8 h at 37 °C, 5% CO₂, washed with PBS, and supplied with fresh pre-warmed medium. Where indicated, FMDV infection was carried out. Live-cell imaging was performed on a label-free microscopy system (SC3000-Pro, Zircon Optics Inc.) with a 40× objective. Time-lapse images were acquired over 4 h. Data were processed with Fiji and ImageJ software [35].

### Scanning electron microscopy (SEM)

Cells grown on coverslips were rinsed with PBS and fixed with electron microscopy fixative (2 h, room temperature). After three washes with 0.1 M phosphate buffer (pH 7.4, 15 min each), samples were post-fixed with 1% osmium tetroxide (1–2 h, dark). Following further buffer washes, dehydration was carried out through a graded ethanol series (30%, 50%, 80%, 90%, 95%, and 100%) and isoamyl acetate (15 min each). Samples were critical-point dried (Hitachi MC1000), mounted on conductive carbon tape, sputter-coated with gold (~ 30 s), and examined using a Hitachi SU8100 SEM.

### Transmission electron microscopy (TEM)

Cell suspensions were mixed with an equal volume of 0.1 M phosphate buffer containing 2.5% glutaraldehyde (1:1), incubated for 10 min, and centrifuged at 1000 × g for 10 min. The pellet was fixed with 2.5% glutaraldehyde for 20 min, centrifuged, and stored at 2–8 °C. After vacuum treatment (2–3 h), samples were assessed for aggregation. Small or loose aggregates were embedded in 0.6–1% agar and cut into pieces; large clumps were directly trimmed. Aggregates were washed at least twice with 0.1 M phosphate buffer (10 min each) and fixed with 1% osmium tetroxide (1–2 h, dark) before TEM processing.

### Definition of viral replication complexes (VRCs)

VRCs were defined as aberrant membrane-bound structures in the cytoplasm of infected cells, surrounded by a single or double membrane and containing filamentous or granular material (viral RNA and proteins). They typically localize near the endoplasmic reticulum or Golgi apparatus.

### Counting criteria

Only membrane structures clearly visible in cellular cross-sections and meeting the above morphological criteria were counted. At least ten distinct cell sections were analyzed per experimental group.

### Exosome isolation and purification

Cell culture supernatant was collected and centrifuged sequentially: 300 × g for 10 min (4 °C), 10,000 × g for 30 min (4 °C), and 100,000 × g for 70 min (4 °C). The final pellet was resuspended in PBS and subjected to a second ultracentrifugation under identical conditions. The purified exosome pellet was resuspended in 200 µL sterile PBS, aliquoted (50 µL per vial), and stored at − 80 °C.

### Ethics statement

All animal experiments were performed by accredited staff at the Center for Animal Experiments, Wuhan University, under protocols approved by the Institutional Animal Care and Use Committee (SKLV-AE2025-007, G2025-037). All animal experiments were performed in accordance with the National Institutes of Health Guide for the Care and Use of Laboratory Animals (8th edition) and the Chinese Regulations for the Administration of Affairs Concerning Experimental Animals (2017 revision). Work with infectious EV71 was conducted in an Animal Biosafety Level-II Laboratory following Institutional Biosafety Committee approval. All samples were inactivated using approved standard operating procedures before removal from containment [36].

### Mouse infection and sample processing

C57BL/6J mice (Hubei BIONT Biological Technology Co., Ltd.) were housed under specific pathogen-free conditions in individually ventilated cages. Six- to eight-week-old mice were anesthetized with isoflurane and inoculated intranasally with 50 µL EV71 wild-type strain (3 × 10⁵ PFU). Control mice received 50 µL MEM. Mice were monitored daily for body weight, clinical signs, and survival; a weight loss to 80% of baseline prompted euthanasia. At least three mice per group were euthanized on days 0, 1, 3, 7, 9, and 10 post-infection. Tissues were weighed, homogenized in PBS, and centrifuged at 5000 rpm for 5 min. Clarified supernatants were stored at − 80 °C.

### Histopathological analysis

Liver and kidney tissues were fixed in 4% paraformaldehyde for 72 h, paraffin-embedded, and sectioned at 3.5 μm. Sections were stained with hematoxylin and eosin (Wuhan Servicebio Technology Co., Ltd.) to evaluate histopathology.

### Statistical analysis

Data are expressed as mean ± SD or mean ± SEM. Differences were assessed using Student’s t-test or one-/two-way ANOVA with Tukey’s multiple comparison test in GraphPad Prism 8. Significance levels: *P < 0.05, **P < 0.01, ***P < 0.001.

For histopathological scoring, liver and kidney injury (e.g., hemorrhage, edema) was evaluated in H&E-stained sections under 10× magnification. Injury distribution was scored across six independent fields based on the percentage of affected tissue area as follows:

- 0 points: no detectable injury;
- 1 point: mild injury (< 25%), slight inflammatory cell infiltration with perivascular cuffing;
- 2 points: moderate injury (25%–50%), increased inflammatory infiltration;
- 3 points: severe injury (50%–75%) involving most of the tissue;
- 4 points: extensive injury (> 75%), inflammatory infiltration throughout the tissue.

## Results

### ARL8B identified as a host factor regulating ssRNA virus replication

**Fig. 1:**
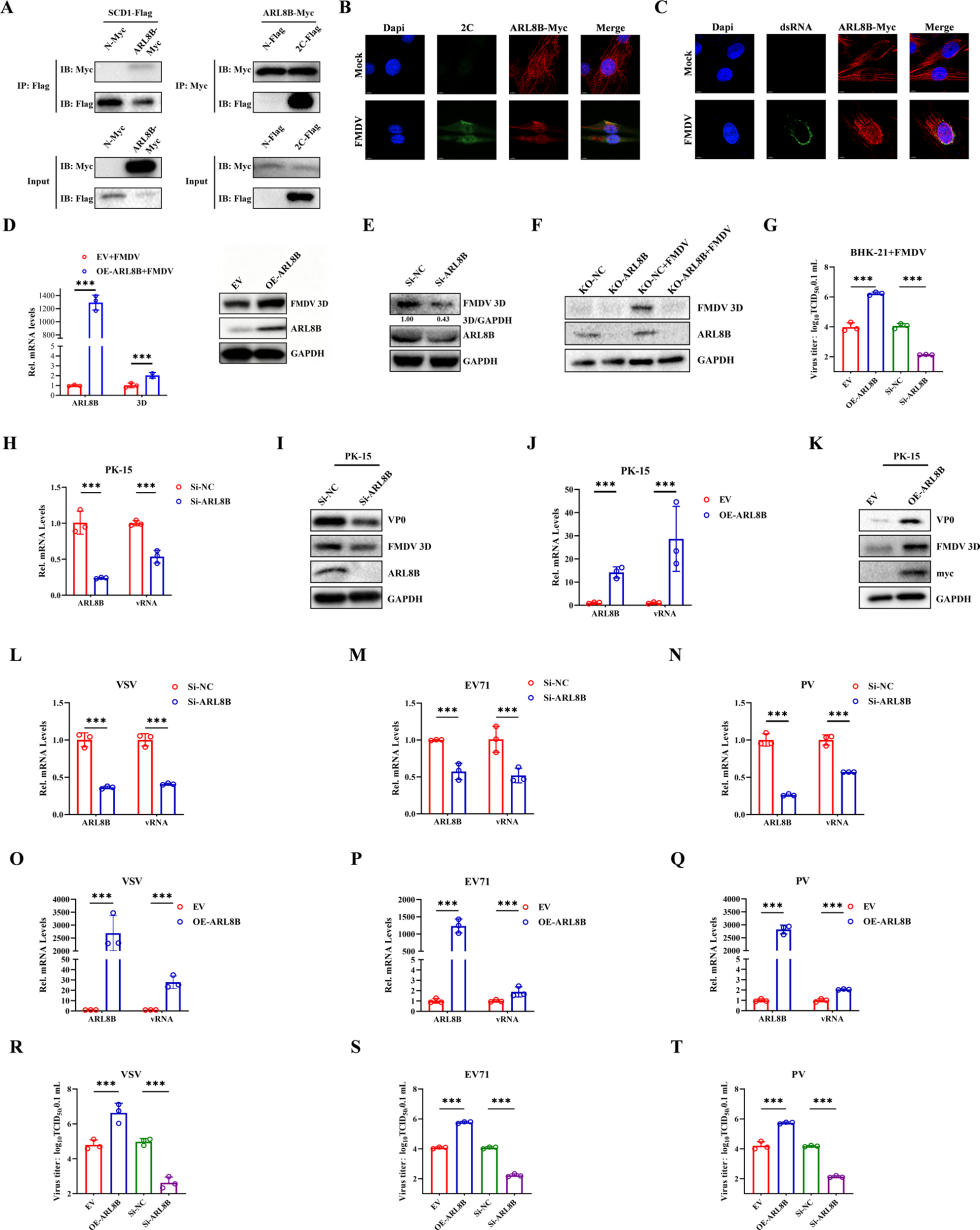
ARL8B constitutes a host factor regulating FMDV replication. (**A**) BHK-21 cells overexpressing Myc-ARL8B were subjected to co-immunoprecipitation using an anti-Myc antibody, followed by Western blotting to detect ARL8B interactions with Flag-SCD1 and viral 2 C protein. (**B**) Immunofluorescence analysis showing subcellular co-localization of viral 2 C protein (green) with ARL8B (red) in FMDV-infected (MOI = 1, 16 h) vs. uninfected control cells. (**C**) Co-localization of dsRNA (green; viral replication intermediate marker) with ARL8B (red) in FMDV-infected cells (MOI = 1, 16 h). (**D**) BHK-21 cells were transfected with empty vector (EV) or ARL8B overexpression plasmid for 24 h, followed by FMDV infection (MOI = 1, 16 h). Viral RNA copy numbers were quantified by reverse transcription quantitative polymerase chain reaction (RT-qPCR, left; *P* < 0.05 vs. EV); ARL8B and viral 3D protein levels were analyzed by Western blotting (right). (**E**) BHK-21 cells were transfected with control siRNA (Si-NC) or ARL8B-specific siRNA (Si-ARL8B) for 24 h, then infected with FMDV (MOI = 1, 16 h). Viral RNA levels were measured by RT-qPCR; ARL8B knockdown efficiency and 3D protein expression were verified by Western blotting (right). (**F**) Comparison of FMDV infection (MOI = 1, 16 h) in ARL8B knockout (KO-ARL8B) and wild-type BHK-21 cells. Western blotting detected the viral nonstructural protein 3D and ARL8B expression levels. GAPDH served as an internal control. (**G**) Vero cells were infected with FMDV(MOI = 1)for 16 h, either with or without ARL8B overexpression plasmids, and with or without ARL8B knockdown plasmids. Viral titers were quantified using the TCID_50_ assay. (**H**–**I**) PK-15 cells with or without ARL8B knockdown were infected with FMDV for 24 h. Viral RNA levels were quantified by RT-qPCR, and protein lysates were analyzed by Western blotting. (**J–K**) PK-15 cells with or without an ARL8B overexpression plasmid were infected with FMDV for 24 h. Viral RNA levels were measured by RT-qPCR, and lysates were analyzed by Western blotting. (**L–N**) Vero cells with or without ARL8B knockdown were infected with VSV (**L**), EV71 (**M**), or poliovirus (**N**) for 24 h. Viral RNA levels were determined by RT-qPCR. (**O–Q**) Vero cells with or without an ARL8B overexpression plasmid were infected with VSV (MOI = 1) (**Q**), EV71 (MOI = 1) (**P**), or poliovirus (MOI = 1) (**Q**) for 24 h. Viral RNA levels were quantified by RT-qPCR. (**R–T**) Vero cells were infected with VSV (**R**), EV71 (**S**), or poliovirus (**T**) for 24 h, either with or without ARL8B overexpression plasmids, and with or without ARL8B knockdown plasmids. Viral titers were quantified using the TCID_50_ assay. Data information: Scale bars, 5 μm. Values represent mean ± SD from three independent experiments. Statistical analysis was performed using t-tests (*n* = 3). *P < 0.05; n.s., not significant

The host protein stearoyl-CoA desaturase 1 (SCD1) directly interacts with FMDV nonstructural protein 2 C within the viral replication complex [35]. Through mass spectrometry–based proteomic analysis, we identified additional host factors that interact with both SCD1 and 2 C [36]. Among these, ARL8B associated with both SCD1 and 2 C in FMDV-infected cells (Fig. 1A). Super-resolution confocal microscopy revealed co-localization of ARL8B with 2 C (Pearson’s *R* = 0.69 ± 0.03 vs. 0.01 ± 0.02 in negative controls; *P* < 0.001) and with the viral replication complex (Pearson’s *R* = 0.58 ± 0.07 vs. 0.10 ± 0.05 in negative controls; *P* < 0.001). Notably, 73.4% ± 6.1% of signals were concentrated in perinuclear regions (Fig. 1B–C). These findings demonstrate that ARL8B is enriched in FMDV viral replication complexes and imply its involvement in viral replication.

To determine the role of ARL8B in FMDV replication, we conducted gain- and loss-of-function assays in BHK-21 cells. ARL8B overexpression significantly enhanced viral replication, increasing viral RNA levels by 2.15-fold (*P* < 0.001) and 3D protein abundance by 2.8-fold at 16 h post-infection (*P* < 0.001) (Fig. 1D). siRNA-mediated knockdown (60% efficiency; Figures S1A, Fig. 1E) reduced viral protein expression by 57% (*P* = 0.002); clustered regularly interspaced short palindromic repeats–CRISPR-associated protein 9 (CRISPR–Cas9) knockout of ARL8B suppressed 3D protein expression by 93% (*P* < 0.001) (Fig. 1F). The results of measuring FMDV titer were consistent with the aforementioned experiments (Fig. 1G). Intriguingly, FMDV infection did not alter ARL8B mRNA or protein abundance throughout the infection period. Although total ARL8B expression remained stable (Figure S1B), its subcellular localization shifted toward perinuclear aggregation (Figure S1C), suggesting spatial regulation of ARL8B activity during viral replication. These results establish ARL8B as a key host regulator of FMDV replication.

We next examined ARL8B function in porcine kidney epithelial (PK-15) cells. ARL8B knockdown significantly reduced viral replication efficiency (Fig. 1H–I), whereas ARL8B overexpression produced dose-dependent increases in viral load (Fig. 1J–K), indicating a conserved role for this host factor across cell types.

The universality of ARL8B-mediated regulation was confirmed through systematic evaluation of three phylogenetically distinct RNA viruses: VSV, poliovirus, and EV71. In Vero cells, ARL8B knockdown reduced viral RNA copy numbers for all three viruses (Fig. 1L–N), whereas ARL8B overexpression enhanced viral replication in a dose-dependent manner (Fig. 1O–Q). The results of measuring VSV, EV71 and PV titer were consistent with the aforementioned experiments (Fig. 1R-T). These results demonstrate that ARL8B holds potential for broad-spectrum applications as a host factor regulating RNA virus replication.

### ARL8B regulates FMDV infection by promoting LD degradation through modulation of the LD-lysosome interaction

To evaluate the effect of ARL8B on LDs, we overexpressed or silenced ARL8B in BHK-21 cells. ARL8B overexpression increased free fatty acid levels, whereas ARL8B knockdown decreased them (Figure S2A). ARL8B upregulation increased LD abundance by 2.71-fold (*P* < 0.001), and ARL8B knockdown reduced LDs by 90% (*P* < 0.001) (Figure S2B–C). Overexpression of ARL8B may simultaneously promote a cyclical process involving lipolysis and lipid synthesis. It accelerates the breakdown of existing lipid droplets, and the fatty acids and other substances released during this breakdown may be reutilized by the cell to form new, additional lipid droplets at sites such as the endoplasmic reticulum (leading to an increase in lipid droplet numbers). This reflects the dynamic equilibrium characteristic of lipid metabolism. Accordingly, ARL8B modulates lipid metabolism, maintaining metabolic homeostasis and facilitating FMDV replication.

**Fig. 2:**
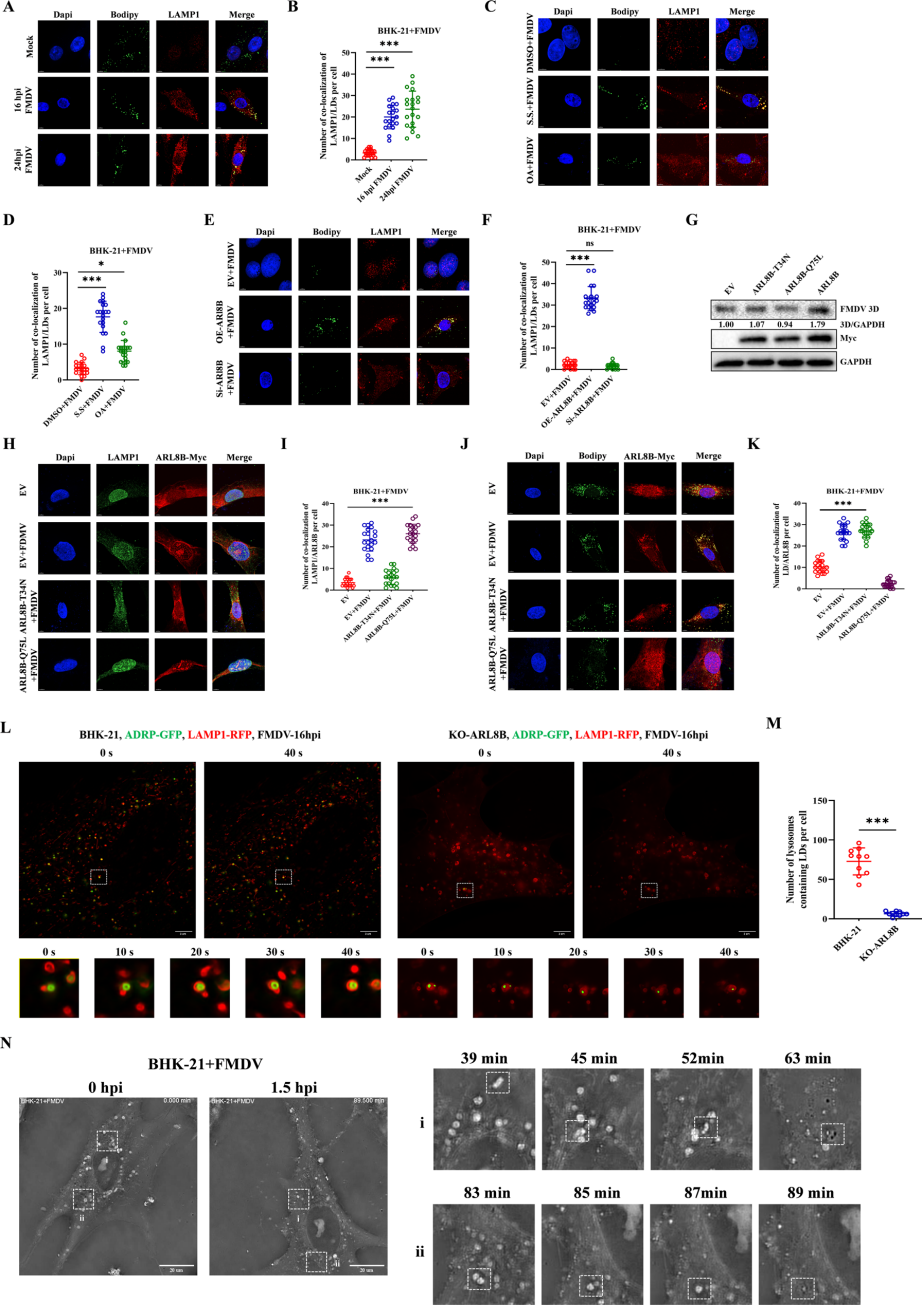
ARL8B modulates LD turnover and directs LD trafficking to lysosomes in FMDV-infected cells. (**A**) BHK-21 cells were mock-treated, FMDV-infected for 16 h, or FMDV-infected for 24 h. LDs were stained with Bodipy (green), lysosomes with LAMP1 antibody (red), and nuclei with 4’,6-diamidino-2-phenylindole (DAPI, blue). (**B**) Quantification of LAMP1/LD co-localization events per cell from 20 cells in three independent experiments, as shown in (**A**). (**C**) BHK-21 cells were treated with dimethyl sulfoxide (DMSO), serum starvation (S.S.), or exogenous oleic acid following FMDV infection (MOI = 1, 16 h). LDs were stained with Bodipy (green), lysosomes with LAMP1 antibody (red), and nuclei with DAPI (blue). (**D**) Quantification of LAMP1/LD co-localization events per cell from 20 cells in three independent experiments, as shown in (**C**). (**E**) BHK-21 cells were treated with EV, ARL8B overexpression, or ARL8B knockdown following FMDV infection (MOI = 1, 16 h). LDs were stained with Bodipy (green), lysosomes with LAMP1 antibody (red), and nuclei with DAPI (blue). (**F**) Quantification of LAMP1/LD co-localization events per cell from 20 cells in three independent experiments, as shown in (**E**). (**G**) BHK-21 cells were transfected with indicated plasmids for 24 h and FMDV-infected (MOI = 1) for 16 h. Lysates were analyzed by Western blotting. (**H**) BHK-21 cells were transfected with indicated plasmids for 24 h and FMDV-infected (MOI = 1) for 16 h. Fixed cells were stained for lysosomes using LAMP1 antibody (green), ARL8B-Myc using anti-Myc antibody (red), and nuclei with DAPI (blue). (**I**) Quantification of LAMP1/ARL8B co-localization events per cell from 20 cells in three independent experiments, as shown in (**H**). (**J**) BHK-21 cells were transfected with indicated plasmids for 24 h and FMDV-infected (MOI = 1) for 16 h. Fixed cells were stained for LDs with Bodipy (green), ARL8B-Myc with anti-Myc antibody (red), and nuclei with DAPI (blue). (**K**) Quantification of LD/ARL8B co-localization events per cell from 20 cells in three independent experiments, as shown in (**J**). (**L**) BHK-21 or KO-ARL8B cells were transfected with indicated plasmids for 24 h, stained for ADRP (LD marker, green) and LAMP1 (lysosomal marker, red), and FMDV-infected (MOI = 1) for 16 h. Cells were analyzed by structured illumination microscopy (SIM). *n* = 10. (**M**) Quantification of LD-containing lysosomes per cell as shown in (**L**). (**N**) BHK-21 cells were infected with FMDV (MOI = 1) for 16 h. LDs (highlighted areas) were visualized using a label-free live-cell microimaging system. Data information: Scale bars, 20 μm. Values represent mean ± SD from three independent experiments. Statistical analysis was performed using t-tests (*n* = 3). *P < 0.05; n.s., not significant

To characterize LD degradation through virus-induced LD–lysosome interactions, we quantified lysosome–LD contacts via lysosome-associated membrane protein 1 (LAMP1)/boron-dipyrromethene (Bodipy 493/503, MCE, HY-D1614) co-localization and assessed FMDV-induced LD degradation under various conditions. FMDV infection alone increased lysosome–LD contact frequency by 6.15-fold (Fig. 2A–B). Enhanced LD degradation was evident during prolonged infection and under serum starvation (Fig. 2C–D). ARL8B overexpression further elevated this interaction to 10.17-fold (Fig. 2E–F). Conversely, ARL8B knockdown reduced lysosome–LD co-localization to baseline levels (0.49-fold vs. control), impairing LD degradation and suppressing FMDV replication.

The guanosine diphosphate (GDP)/GTP cycle of ARL8B reportedly mediates lysosome-dependent LD turnover [2]. To elucidate the mechanism by which ARL8B regulates LD degradation and its role in FMDV replication, we examined the utility of ARL8B as a molecular switch for LD breakdown. A previous study identified two conserved residue mutants of ARL8B: a GTP-locked form (Q75L) and a GDP-locked form (T34N) [27]. Co-immunoprecipitation experiments confirmed that these mutations did not disrupt ARL8B oligomerization (Figure S2D–E). However, functional assays revealed that overexpression of either mutant in BHK-21 cells failed to enhance FMDV replication (3D protein levels: ARL8B-T34N = 1.07-fold, ARL8B-Q75L = 0.94-fold vs. control; Fig. 2G), demonstrating that GTP/GDP cycling is essential for ARL8B regulation of host lipid metabolism. Subcellular localization analysis showed distinct patterns for the two mutants. The GTP-bound Q75L mutant preferentially localized to lysosomes (*P* < 0.001), whereas the GDP-bound T34N mutant accumulated on LDs (*P* < 0.001) (Fig. 2H–K). During FMDV infection, ARL8B may control lipid release by modulating lysosome–LD interactions; its GTPase activity—rather than its oligomerization—is the key determinant of function.

To visualize the interaction between LDs and lysosomes at high temporal resolution, we utilized structured illumination microscopy at 50 ms frame^− 1^ resolution. Images showed that 87% ± 4% of LDs (adipose differentiation-related protein [ADRP]–green fluorescent protein [GFP]) in wild-type cells (*n* = 50) formed sustained contacts with lysosomes (LAMP1-mCherry) lasting at least 30 s. These interactions followed a defined sequence: initial docking (0–5 s) was characterized by lysosomal surface expansion to 1.78-fold the original area, followed by peripheral elongation along LDs (5–15 s), and culminating in complete LD encapsulation through lysosomal membrane fusion (15–30 s). ARL8B knockout cells displayed severe defects: lysosomes underwent pathological expansion (5.62-fold increase in surface area; *P* < 0.001 vs. wild-type), and only 9% successfully encapsulated LDs. Most interactions stalled at the docking phase and were accompanied by lysosomal structural abnormalities (Fig. 2L–M). Super-resolution imaging thus revealed clear differences between wild-type and ARL8B-deficient cells. These findings demonstrate that ARL8B is essential for proper lysosomal remodeling during LD interaction; its role in maintaining lysosomal structural integrity directly influences host cell LD degradation.

Exploiting the intrinsically high refractive index of LDs, we utilized label-free live-cell imaging to track dynamic LD degradation in FMDV-infected BHK-21 cells (multiplicity of infection [MOI] = 1) over 1.5 h (30 s frame^− 1^). Quantitative phase analysis demonstrated that FMDV infection induced progressive cleavage of parental LDs (> 2 μm) into secondary LDs (0.5–1 μm), which were encapsulated by autophagosomes to initiate lipophagy (Fig. 2N). This sequential process—LD fission followed by autophagic clearance—illustrates how FMDV transforms static LDs into transient lipid supply units.

LDs play dual roles in viral replication, serving as an energy reservoir and a regulatory platform [37]. To investigate the relationship between viral production and ARL8B, we quantified co-localization of the FMDV structural protein VP0 with LDs as an indicator of viral assembly. FMDV infection (MOI = 1) promoted VP0–LD co-localization; the extent of overlap gradually increased, confirming that LDs comprise critical sites for FMDV assembly and synthesis (Figure S2F–G). ARL8B overexpression and serum starvation further enhanced VP0–LD co-localization, indicating increased viral production (Figure S2F–I). These results suggest that ARL8B facilitates viral synthesis by promoting host LD degradation. The resulting increase in lipid availability creates a favorable metabolic environment for FMDV replication, demonstrating a strong positive correlation between viral replication and host LD turnover.

### ARL8B-induced lipophagy facilitates lipid release to support FMDV replication complex assembly

**Fig. 3:**
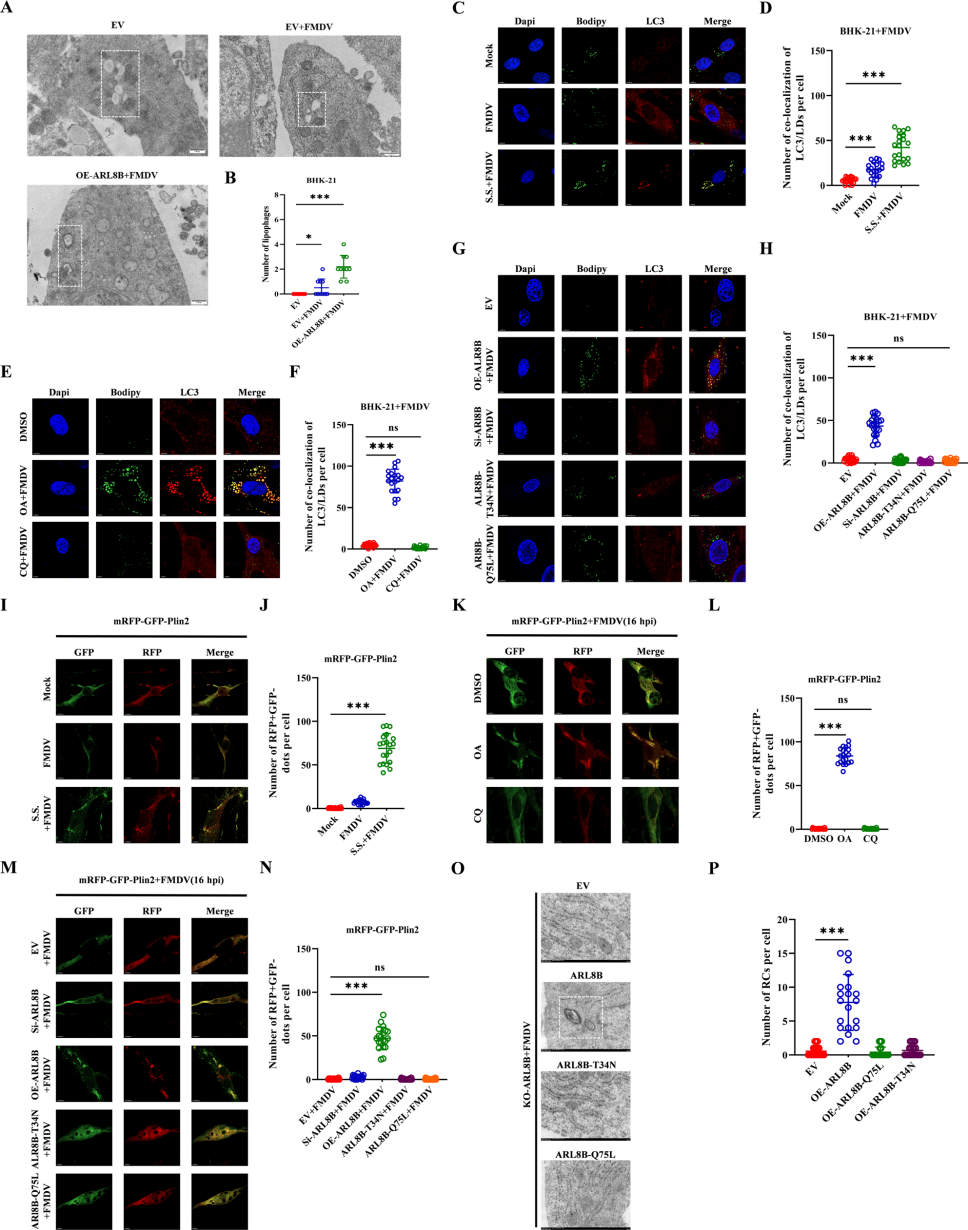
ARL8B-induced lipophagy enhances lipid catabolism during FMDV infection. (**A**) Transmission electron microscopy (TEM) images of lipid droplets (LDs) in BHK-21 cells infected with FMDV (MOI = 1, 16 h) or overexpressing ARL8B for 24 h. White boxes indicate LDs, and the magnified regions show LD morphology. (**B**) Quantification of lipophagic events from 10 randomly selected fields of view. (**C**) BHK-21 cells were subjected to the indicated treatments (mock, FMDV infection [MOI = 1, 16 h], or S.S.), then fixed and stained with anti-LC3 antibody (red) to label autophagic vesicles, Bodipy (green) to label LDs, and DAPI (blue) to stain nuclei. (**D**) Quantification of LC3/LD co-localization events per cell from 20 cells across three independent experiments, as shown in (**C**). (**E**) BHK-21 cells were treated as indicated (DMSO [control], S.S., exogenous oleic acid [OA], or CQ) following FMDV infection (MOI = 1, 16 h), then fixed and stained with anti-LC3 antibody (red), Bodipy (green), and DAPI (blue). (**F**) Quantification of LC3/LD co-localization events per cell from 20 cells across three independent experiments, as shown in (**E**). (**G**) BHK-21 cells were transfected with the indicated constructs (EV, ARL8B overexpression, ARL8B knockdown, or ARL8B point mutants), followed by FMDV infection (MOI = 1, 16 h). Cells were fixed and stained with anti-LC3 antibody (red), Bodipy (green), and DAPI (blue). (**H**) Quantification of LC3/LD co-localization events per cell from 20 cells across three independent experiments, as shown in (**G**). (**I**,** K**,** M**) BHK-21 cells expressing mRFP-GFP-Plin2 were treated for 48 h under the indicated conditions and observed by confocal microscopy. (**J**,** L**,** N**) Quantification of RFP/GFP co-localization events per cell from 20 cells across three independent experiments, corresponding to (**I**,** K**,** M**). (**O**) TEM images of BHK-21 cells infected with FMDV (MOI = 1, 16 h) or overexpressing ARL8B, ARL8B-T34N, or ARL8B-Q75L for 24 h, showing viral replication complex morphology (highlighted in white boxes). (**P**) Quantification of replication complexes per cell from 20 cells across three independent experiments. Data information: Scale bars, 500 nm (**A**,** O**) or 5 μm (others). Values represent mean ± SD from three independent experiments. Statistical analysis was performed using t-tests (*n* = 3). *P < 0.05; n.s., not significant

Microlipophagy and macrolipophagy occur downstream of lysosome-dependent LD degradation [38]. As described above, FMDV infection promotes LD breakdown, triggering lipophagy. To examine FMDV-induced lipophagy activity under various conditions, we assessed LD autophagy via transmission electron microscopy (TEM). FMDV infection alone did not cause extensive autophagic encapsulation of LDs (Fig. 3A). In contrast, ARL8B overexpression strongly induced lipophagy (Fig. 3A–B). To examine FMDV-mediated lipophagy under different conditions, we quantified co-localization between the autophagosome marker LC3 and the LD marker Bodipy as a measure of lipophagic activity. Changes in co-localization number were used to assess lipophagic strength by confocal fluorescence microscopy. Although immunofluorescence detection showed a modest but significant increase in LD–autophagosome co-localization after FMDV infection (*P* < 0.05) (Fig. 3C–D), implying that FMDV alone only triggers limited lipophagy under basal conditions. Both oleic acid treatment and serum starvation substantially enhanced lipophagy, whereas ARL8B knockdown and addition of the autophagy inhibitor chloroquine (CQ) significantly suppressed it (Fig. 3C–F). In contrast, ARL8B overexpression strongly increased lipophagic activity (7.49-fold; *P* < 0.001) (Fig. 3G–H), establishing ARL8B as a critical regulatory factor in FMDV-induced lipophagy. The GTP-locked (Q75L) and GDP-locked (T34N) variants of ARL8B failed to enhance lipophagy. These results establish ARL8B-mediated lipophagy as a key mechanism for mobilizing lipid resources to support FMDV replication.

Using a monomeric red fluorescent protein (mRFP)–GFP–perilipin 2 (Plin2) dual-fluorescence reporter system to avoid artifacts associated with LC3 antibody staining, we precisely tracked the lipophagy process through fluorescence conversion. This system distinguished early autophagosomes (GFP+/RFP+ yellow signals under pH-neutral conditions) from late degradation stages (RFP + GFP− red signals at pH < 5.0), corresponding to Plin2-containing autophagosomes and autolysosomal fusion events, respectively. ARL8B overexpression, exogenous oleic acid treatment, and serum starvation all promoted lipophagy, whereas CQ treatment and ARL8B knockdown inhibited it. Late-stage lipophagy (red puncta only) was often localized to the perinuclear region, suggesting that lipophagic activity originates near the nucleus and expands outward. The results closely paralleled outcomes from LC3/Bodipy co-localization assays (Fig. 3I–N), confirming the reliability of this approach for quantifying lipophagic flux. Our findings indicate that ARL8B dynamically regulates both lysosome–LD contact and LC3-mediated lipophagosome formation through its GTP/GDP cycle. This ARL8B-dependent control of LD degradation and subsequent LD phagocytosis fundamentally reprograms host lipid metabolism, supplying the lipid substrates necessary for assembly of the FMDV replication complex.

Functional assays in ARL8B knockout cells demonstrated that complementation with wild-type ARL8B restored lipophagy and replication complex formation, whereas GTPase-deficient mutants (T34N and Q75L) severely impaired these processes (Fig. 3O–P). Quantitative analysis showed 78%–82% reductions in replication complex formation within cells expressing the mutants relative to controls, confirming that ARL8B enzymatic cycling is essential for lysosome-mediated LD degradation, fatty acid release, and viral membrane remodeling. These results clarify how FMDV exploits host metabolic pathways through ARL8B-mediated lipophagy; they also identify this mechanism as a promising target for broad-spectrum antiviral development.

### ARL8B-mediated autophagic activation promotes FMDV replication

**Fig. 4:**
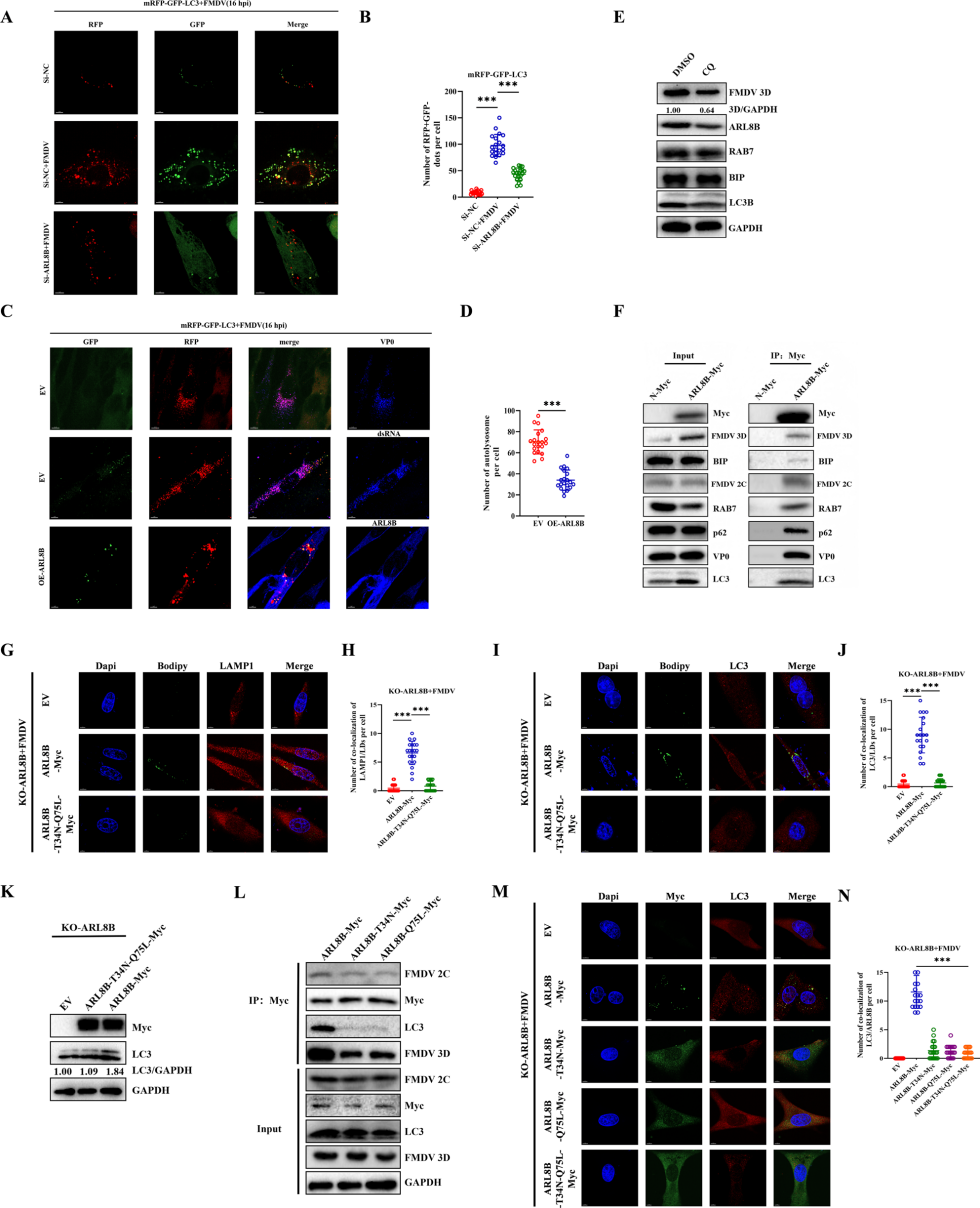
ARL8B-mediated autophagic activation promotes FMDV replication. (**A**) BHK-21 cells expressing mRFP-GFP-LC3 (48 h post-transfection) were infected with FMDV (MOI = 1, 16 h) or transfected with an ARL8B overexpression plasmid, then fixed and imaged by confocal microscopy. (**B**) Quantification of RFP/GFP co-localization events per cell from 20 cells across three independent experiments. (**C**) BHK-21 cells expressing mRFP-GFP-LC3 were treated for 48 h under the indicated conditions, followed by staining with antibodies against dsRNA, VP0, and ARL8B (blue). (**D**) Quantification of autolysosomes per cell from 20 cells across three independent experiments. (**E**) FMDV-infected BHK-21 cells (MOI = 1, 16 h) were treated with CQ (20 µM, 6 h); cell lysates were analyzed by Western blotting. (**F**) Co-immunoprecipitation analysis of ARL8B interactions with viral proteins (2 C, 3D, VP0) and host proteins (Rab7, BIP, p62, LC3). (**G**) KO-ARL8B cells were treated as indicated and then fixed. Lysosomes were stained with LAMP1 antibody (red), LDs with Bodipy (green), and nuclei with DAPI (blue). (**H**) Quantification of LAMP1/LD co-localization events per cell from 20 cells across three independent experiments. (**I**) Cells were treated as indicated and fixed. Autophagic vesicles were stained with LC3 antibody (red), LDs with Bodipy (green), and nuclei with DAPI (blue). (**J**) Quantification of LC3/LD co-localization events per cell from 20 cells across three independent experiments. (**K**) BHK-21 cells were transfected with an empty vector, ARL8B wild-type, or ARL8B double-point mutant plasmid for 24 h, followed by FMDV infection (MOI = 1, 16 h). Cell lysates were analyzed by Western blotting. (**L**) Co-immunoprecipitation analysis of ARL8B point mutants to assess interactions with 2 C, 3D, and LC3 proteins. Note: Under the experimental conditions employed, LC3-I and LC3-II were not completely separated. Changes in the overall band intensity were used to assess autophagic flux and interactions. (**M**) Cells were treated under the indicated conditions and fixed. Autophagic vesicles were stained with LC3 antibody (red), ARL8B and its mutants with anti-Myc antibody (green), and nuclei with DAPI (blue). (**N**) Quantification of LC3/ARL8B co-localization events per cell from 20 cells across three independent experiments. Data information: Scale bars, 5 μm. Values represent mean ± SD from three independent experiments. Statistical analysis was performed using t-tests (*n* = 3). *P < 0.05; n.s., not significant

Building on the established lysosomal anchoring function of ARL8B [25] and its demonstrated role in lipophagy, we investigated how ARL8B influences host cell autophagy. We utilized a pH-sensitive RFP-GFP-LC3 dual-fluorescence reporter system (pLVX-RFP-GFP-LC3 lentivirus (Han Heng Biotechnology), MOI = 5) to monitor autophagic flux in FMDV-infected BHK-21 cells. Fluorescence imaging revealed that FMDV infection accelerated autophagosome maturation, producing an 11.49-fold increase (*P* < 0.001) in autophagolysosome formation (GFP−/RFP + red puncta) relative to autophagosomes (GFP+/RFP+ yellow puncta) (Fig. 4A–B). Spatial mapping showed that 78% of autophagolysosomes (*n* = 20) localized to perinuclear regions. ARL8B knockdown reduced autophagolysosome formation efficiency by 56% (*P* < 0.001), confirming ARL8B’s involvement in regulating host cell autophagy flux. Interestingly, in ARL8B-knockdown cells, the loss of FMDV-induced GFP-LC3 punctate signals is more complete, suggesting that ARL8B deficiency may alter autophagic flux kinetics or terminal degradation steps in a complex manner.

To delineate the relationship between FMDV-induced autophagy and ARL8B function, we performed high-resolution co-localization studies using viral markers (VP0, double-stranded RNA [dsRNA]) and autophagy indicators (LC3). Quantitative analysis revealed strong associations of viral components with autophagolysosomes (LAMP1+/LC3+), with Pearson correlation coefficients of *R* = 0.78 for VP0 and *R* = 0.86 for dsRNA (*n* = 20 cells); 87% of co-localization events were concentrated in perinuclear regions (Fig. 4C–D). ARL8B overexpression induced extensive autophagolysosome remodeling, decreasing their number by 43% while increasing their individual volumes 3.1-fold, consistent with enhanced autophagic flux. Multicolor imaging showed that 76% of autophagolysosomes co-localized with endoplasmic reticulum markers (ERp72; *R* = 0.84); 72% of these sites were also associated with the Golgi marker GM130, forming specialized endoplasmic reticulum–Golgi–lysosome membrane contact sites (Figure S3A). TEM ultrastructural analysis confirmed lysosome–endoplasmic reticulum interactions (< 30 nm contact distance) after FMDV infection (Figure S3B), indicating that widespread autophagic activity in host cells primarily occurs at the endoplasmic reticulum and Golgi apparatus. Pharmacological inhibition with CQ reduced FMDV 3D protein expression by 64% and decreased ARL8B protein levels (Fig. 4C-D). Expression of the lysosomal transport–associated proteins Rab7 and BIP remained unchanged during CQ treatment, suggesting that the ARL8B–Rab7–BIP complex retains structural integrity during autophagy inhibition (Fig. 4E). However, CQ-mediated inhibition of autophagy strongly suppressed FMDV replication. These findings establish autophagy as essential for efficient FMDV replication; ARL8B constitutes the central regulator. Comprehensive interaction screening revealed that ARL8B binds viral proteins (structural VP0; nonstructural 3D and 2 C) and host autophagy machinery (LC3, p62), as well as lysosomal transport components (Rab7, BIP) (Fig. 4F). This unique network of interactions positions ARL8B as a molecular hub integrating viral replication with host autophagy pathways.

Through comprehensive functional validation of Q75/T34 double mutants in ARL8B, we established that these conserved residues function as an essential molecular switch governing ARL8B–LC3 interactions and subsequent lipophagy activation. Double and single mutants displayed identical loss-of-function phenotypes, completely lacking LD degradation and lipophagic activity (Fig. 4G–J). Exogenous overexpression of the double mutant in ARL8B knockout cells failed to restore autophagic flux (Fig. 4K). Biochemical and imaging analyses demonstrated that mutation of either residue alone abolished ARL8B–LC3 binding, as confirmed by co-immunoprecipitation assays (Fig. 4L), and eliminated their cellular co-localization (Fig. 4M–N). These findings establish a requirement for intact Q75 and T34 residues in mediating ARL8B–LC3 interactions, which regulate a critical checkpoint in autophagosome maturation. The non-redundant nature of these residues underscores the precision with which FMDV exploits the ARL8B GTPase domain to hijack host autophagy machinery, redirecting lipid metabolism to create specialized viral replication niches while evading cellular surveillance.

### ARL8B facilitates RNA virus egress via the lysosomal–exosome transport pathway

**Fig. 5:**
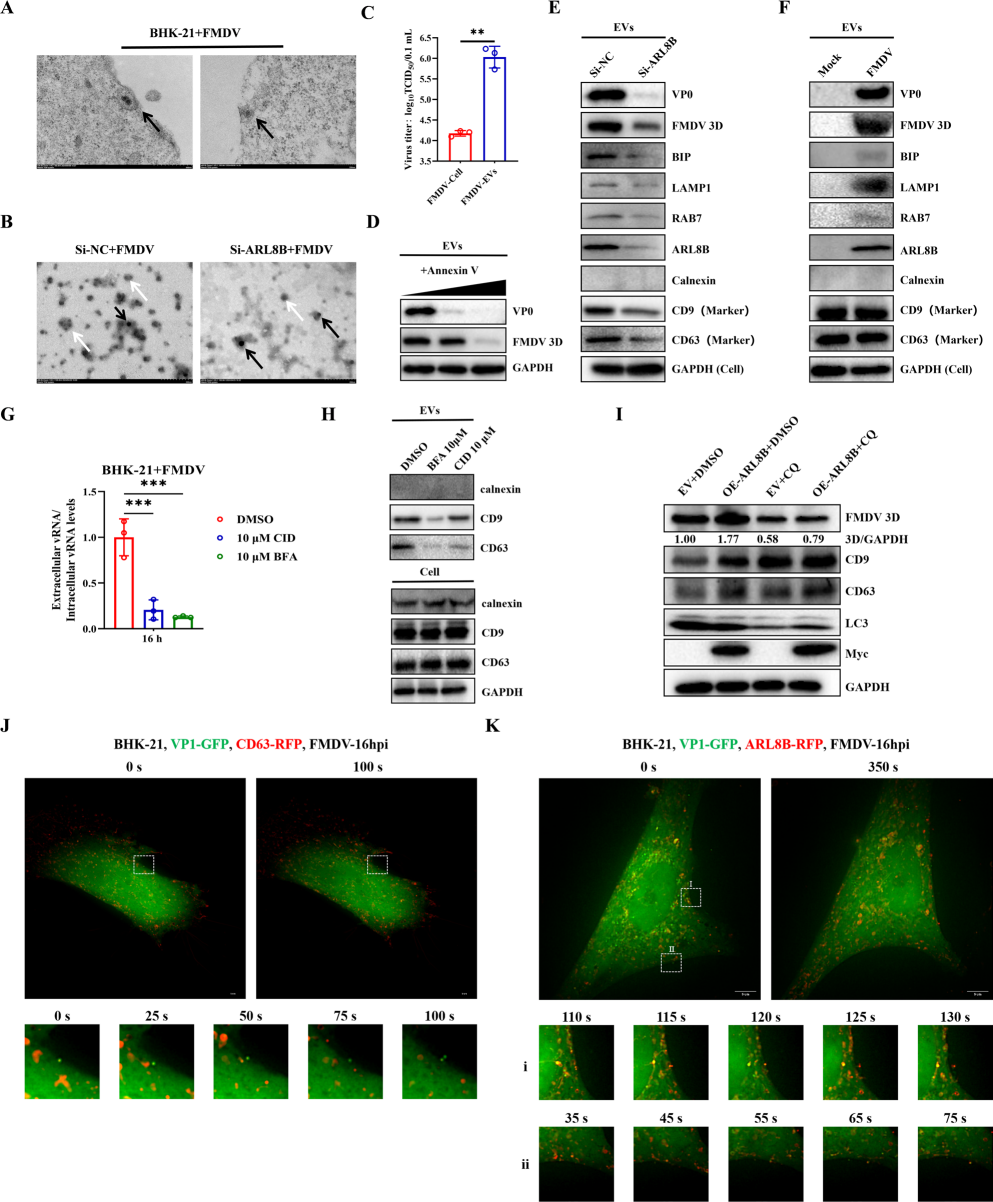
ARL8B facilitates FMDV egress via the lysosomal–exosome transport pathway. (**A**) TEM of BHK-21 cells infected with FMDV (MOI = 1, 16 h) revealed vesicles containing viral particles (black arrowheads; diameter 80–120 nm). Samples were fixed with 2.5% glutaraldehyde and 1% osmium tetroxide, then stained with uranyl acetate. (**B**) TEM images of extracellular vesicles (EVs) from ARL8B knockdown (Si-ARL8B) BHK-21 cells 24 h after FMDV infection. Black arrows indicate vesicles containing viral particles; white arrows indicate EVs lacking viral particles. (**C**) Extracted EVs (FMDV-EVs) and cell lysates (FMDV-Cell) were added separately to BHK-21 cultures, and viral titers were measured 48 h post-infection. (**D**) BHK-21 cells were infected after co-incubation with EVs and increasing concentrations of Annexin V. Cell lysates were analyzed by Western blotting. (**E**) EVs extracted from FMDV-infected or uninfected BHK-21 cells were analyzed by Western blotting. (**F**) EVs extracted from wild-type or ARL8B-knockdown BHK-21 cells infected with FMDV (MOI = 1) were analyzed by Western blotting. (**G**) Ratios of extracellular to intracellular viral RNA (vRNA) were determined by RT-qPCR after treatment with BFA or CID. (**H**) EVs extracted after treatment with BFA or CID were analyzed by Western blotting. (**I**) After CQ treatment and ARL8B overexpression, lysates were analyzed by Western blotting to detect expression of 3D, CD9, CD63, and LC3. (**J**) BHK-21 cells and (**K**) KO-ARL8B cells were transfected with the indicated plasmids for 24 h, stained for VP1 (green) and CD63 (exosome marker, red), and infected with FMDV (MOI = 1) for 16 h. Cells were visualized by SIM. Data information: Scale bars, 500 nm (**A**,** B**) or 3 μm (others). Values represent mean ± SD from three independent experiments. Statistical analysis was performed using t-tests (*n* = 3). *P < 0.05; n.s., not significant

There is increasing evidence that RNA viruses exploit EVs for dissemination, utilizing apoptotic vesicles and Golgi–lysosomal pathways [39]. Our TEM analysis confirmed that mature FMDV particles are secreted via EVs (Fig. 5A), which exhibited distinct morphological features relative to endocytosed viral particles [40]. Negative staining revealed characteristic cup-shaped exosomes (190.3 ± 73.7 nm in diameter) containing electron-dense particles (29.8 ± 3.1 nm in diameter) that matched the size of purified FMDV virions (30.5 ± 2.8 nm). Strikingly, ARL8B knockdown significantly increased the proportion of empty capsids within EVs, from 32 ± 4% in wild-type cells to 77 ± 6% (Fig. 5B), demonstrating ARL8B’s critical role in regulating FMDV extracellular transport through EVs. Freeze–thaw–reinfection experiments verified the presence of FMDV particles within EVs (Figure S4A) and demonstrated that EVs are highly fragile, undergoing rapid degradation in vitro. Functional assays showed that EV-mediated infection (0.5 µg µL⁻^1^) yielded significantly higher viral titers compared with free virus released by cell lysis at 24 h post-infection (Fig. 5C), indicating superior infection efficiency through EV-mediated intercellular transmission. This process required phosphatidylserine-mediated membrane fusion, given that Annexin V (MCE) pretreatment (10 µg mL^− 1^, 1 h) reduced exosome-mediated infection by 90% (Fig. 5D). These findings suggest that FMDV hijacks the host exosomal pathway through ARL8B-dependent mechanisms; phosphatidylserine exposure serves as a key determinant of vesicle infectivity. EV characterization confirmed their exosomal identity by demonstrating strong enrichment of canonical markers (CD63, CD81, TSG101) and minimal calnexin contamination (< 5% of cellular levels), fully consistent with Minimal Information for Studies of Extracellular Vesicles 2018 (MISEV2018) standards for BHK-21–derived exosomes (Fig. 5E). These EVs contained both structural (VP0) and nonstructural (3D) viral proteins, as well as intact capsids (Figs. 5E, S4A). Additionally, the presence of LAMP1 and ARL8B in FMDV-loaded EVs, along with the specific incorporation of Rab7 and BIP in an ARL8B-dependent manner (Fig. 5E). This coordinated transport was supported by strong extracellular ARL8B–BIP co-localization (*R* = 0.69; Figure S4B). ARL8B knockdown substantially impaired EV production while simultaneously reducing exosomal levels of viral components (3D, VP0), lysosomal transport machinery (Rab7, LAMP1), and quality-control factors (BIP) (Fig. 5F). These findings demonstrate that ARL8B functions as a master regulator integrating three critical modules of vesicular trafficking: Rab7-mediated membrane fusion, BIP-dependent protein translocation, and LAMP1-guided quality control. Comprehensive disruption of all three systems upon ARL8B depletion explains the severe impairment of both EV biogenesis and viral secretion. These observations reveal an elegant host–pathogen adaptation in which FMDV commandeers the lysosomal export pathway through ARL8B’s unique ability to synchronize multiple trafficking systems, thereby enhancing viral dissemination.

Targeted pharmacological inhibition of key cellular trafficking pathways clarified the secretory routes exploited by FMDV. Blockade of lysosomal trafficking with CID-1,067,700 (CID) strongly suppressed both viral replication and particle release (Figure S4C), suggesting that whereas approximately 40% of virions utilize conventional secretion, the majority rely on lysosomal export. Similarly, disruption of endoplasmic reticulum–to–Golgi transport using brefeldin A (BFA) significantly impaired viral replication without altering ARL8B expression (Figure S4D), indicating that FMDV utilizes the endoplasmic reticulum–Golgi secretory pathway; ARL8B is not directly involved in this route. Both inhibitors substantially reduced extracellular viral loads and EV release (Fig. 5G–H), confirming parallel exploitation of secretory pathways. Further mechanistic insights were obtained through autophagy inhibition using CQ (20 µM, 6 h) (CCK8, Figure S4E–G), which impaired exosome biogenesis by alkalinizing lysosomal pH and thus reducing CD9/CD63 expression (Fig. 5I). The ability of ARL8B overexpression to rescue CQ-mediated suppression highlights ARL8B’s essential role in maintaining lysosomal function to support exosome maturation. These findings confirm that FMDV employs a dual secretion strategy, hijacking both classical secretory and lysosomal export pathways. ARL8B serves as the central regulator of the dominant lysosome-dependent route through its ability to sustain autophagic flux and vesicular trafficking competence.

Time-course analysis revealed that extracellular FMDV release was closely correlated with replication kinetics, displaying progressive accumulation (Figure S4H). Concurrent treatment with CID, BFA, and ARL8B knockdown effectively suppressed extracellular EV accumulation. A Gaussia luciferase secretory reporter system (pLVX-SecGluc) quantified the impact of FMDV on host secretory pathways: Golgi-dependent secretion increased by 2.1-fold compared with controls (*P* < 0.01). CID (10 µM) and BFA (10 µM) reduced Golgi secretory activity by 68% and 72%, respectively (*P* < 0.001) (Figure S4I). Combined ARL8B knockout and BFA treatment nearly abolished extracellular viral RNA, demonstrating that simultaneous inhibition of both secretion pathways effectively blocks FMDV release (Figure S4J). These findings highlight FMDV’s active manipulation of classical secretion to facilitate viral release.

Super-resolution imaging captured the dynamic release of virus-loaded exosomes (CD63+/VP1+) through membrane protrusions (Fig. 5J–K). Single-particle tracking confirmed ARL8B’s direct involvement at exosomal release sites (Fig. 5K). Moreover, exosome secretion occurred throughout the cell rather than at specific localized regions. SEM revealed that FMDV infection induced a 3.3-fold increase in exosome diameter (205.9 ± 68.5 nm vs. control 62.9 ± 30.8 nm), whereas ARL8B knockdown restored exosome size to baseline levels (57.6 ± 24.0 nm, *P* < 0.001) while reducing the abundance of those exosomes. These findings highlight the dual roles of ARL8B in regulating the quantity and diameter of virus-carrying exosomes (Figure S4K–M). Furthermore, exosome diameter increased according to distance from the cell nucleus, indicating progressive membrane fusion events during the secretion process (Figure S4N).

To determine whether ARL8B regulates RNA virus replication through a similar mechanism in other RNA viruses, such as EV71 and VSV, we utilized the same experimental approach. Dual-fluorescence imaging (RFP-GFP-Plin2 for lipophagy; RFP-GFP-LC3 for autophagic flux) showed that ARL8B depletion impaired autophagosome–lysosome fusion, as reflected by decreased yellow puncta (Figure S4O–R). These results highlight ARL8B’s central role in coordinating both specialized lipophagy and canonical autophagy pathways to establish a microenvironment conducive to RNA virus replication. Mechanistic studies then revealed that pharmacological inhibition of secretory pathways (CID or BFA treatment) or ARL8B knockdown similarly suppressed extracellular release of EV71 and VSV (Figure S4S–X). Taken together, these findings establish that ARL8B’s regulation of viral particle release through modulation of secretory pathways is not limited to FMDV—it extends across multiple single-stranded RNA virus families.

Collectively, our results imply that FMDV strategically co-opts both the classical Golgi secretory pathway and ARL8B-regulated lysosomal transport for extracellular release. The virus orchestrates this dual exploitation approach by (1) enhancing conventional secretion through coat protein–dependent mechanisms and (2) remodeling exosome biogenesis via ARL8B-mediated control of vesicular size and membrane dynamics, thus generating specialized extracellular carriers for efficient viral dissemination.

### Small-molecule compounds targeting ARL8B inhibit single-stranded RNA virus replication

**Fig. 6:**
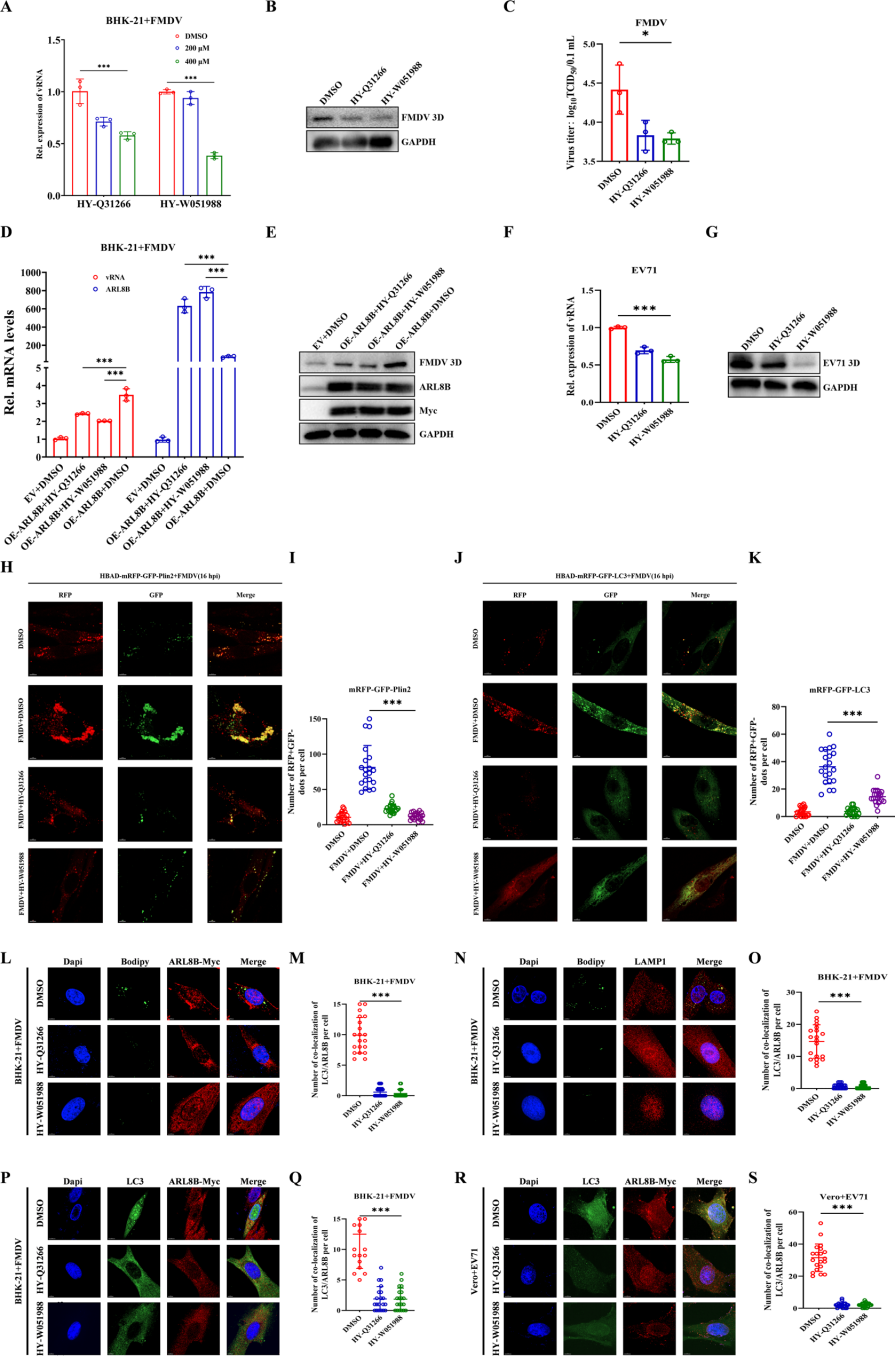
Small-molecule compounds targeting ARL8B inhibit single-stranded RNA virus replication. (**A–C**) BHK-21 cells were treated with varying concentrations of HY-Q31266 or HY-W051988, then infected with FMDV (MOI = 1, 16 h). Viral RNA levels were quantified by RT-qPCR, lysates were analyzed by Western blotting, and viral titers were determined by tissue culture infectious dose 50% (TCID_50_) assay. (**D–E**) BHK-21 cells were transfected or not with an ARL8B overexpression plasmid for 24 h, then treated with HY-Q31266 or HY-W051988 and infected with FMDV (MOI = 1, 16 h). Viral RNA levels were measured by RT-qPCR; lysates were analyzed by Western blotting. (**F–G**) Vero cells were treated with HY-Q31266 or HY-W051988 and infected with EV71 for 24 h. Viral RNA levels were determined by RT-qPCR, and protein expression was analyzed by Western blotting. (**H**) Vero cells expressing mRFP-GFP-Plin2 were treated for 48 h under the conditions indicated at left, then fixed and visualized by confocal microscopy. (**I**) Quantification of RFP/GFP co-localization events per cell from 20 cells across three independent experiments, as shown in (**H**). (**J**) Vero cells expressing mRFP-GFP-LC3 were treated for 48 h under the indicated conditions, fixed, and observed by confocal microscopy. (**K**) Quantification of RFP/GFP co-localization events per cell from 20 cells across three independent experiments, as shown in (**J**). (**L**) Cells were treated under the indicated conditions, then fixed and stained for lipid droplets (Bodipy, green), ARL8B (anti-Myc antibody, red), and nuclei (DAPI, blue). (**M**) Quantification of LD/ARL8B co-localization events per cell from 20 cells across three independent experiments, as shown in (**L**). (**N**) Cells were treated under the indicated conditions, then fixed and stained for lipid droplets (Bodipy, green), lysosomes (LAMP1 antibody, red), and nuclei (DAPI, blue). (**O**) Quantification of LD/LAMP1 co-localization events per cell from 20 cells across three independent experiments, as shown in (**N**). (**P**,** R**) Cells were treated under the indicated conditions, then fixed and stained for autophagic vesicles (LC3 antibody, green), ARL8B (anti-Myc antibody, red), and nuclei (DAPI, blue). (**Q**,** S**) Quantification of LC3/ARL8B co-localization events per cell from 20 cells across three independent experiments, as shown in (**P**,** R**). Data information: Scale bars, 5 μm. Values represent mean ± SD from three independent experiments. Statistical analysis was performed using t-tests (*n* = 3). *P < 0.05; n.s., not significant

Building upon the conserved structural architecture of ARL8B’s functional domain (Q34/T75), we implemented a structure-based virtual screening strategy against the HY-L901 MCE 50 K compound library to identify potential antiviral agents. Molecular docking simulations utilizing the predicted ARL8B conformation (AlphaFold ID: AF-A0A1U7Q8M2-F1) identified two compounds, HY-W051988 and HY-Q31266 (Figure S5A), both of which demonstrated favorable binding energetics (Figure S5B-C). These compounds exhibited excellent safety profiles, with half-maximal inhibitory concentration (CC_50_) values exceeding 10 µM (Figure S5D), indicating negligible cytotoxicity. Pharmacological evaluation revealed dose-dependent inhibition of FMDV replication, such that 400 nM concentrations of HY-W051988 and HY-Q31266 reduced viral RNA loads by 38.4% and 57.9%, respectively (Fig. 6A–C). The antiviral effect was specifically mediated through ARL8B targeting, given that viral replication was significantly rescued (*P* < 0.01) by wild-type ARL8B overexpression in compound-treated cells (Fig. 6D–E). The broad-spectrum potential of these inhibitors was confirmed by their similar efficacy against EV71, reducing viral RNA by 31%–43% (Fig. 6F–G), which established ARL8B as a conserved host factor across multiple single-stranded RNA viruses.

Mechanistic investigations demonstrated that both compounds disrupt ARL8B’s multifaceted role in viral replication through three convergent actions: (1) impairment of autophagic flux, as indicated by altered LC3/Plin2 dynamics in dual-fluorescence tracking assays (Fig. 6H–K); (2) suppression of ARL8B–LD co-localization (Fig. 6L–M) and LD degradation (Fig. 6N–O), resulting in an 84.5% reduction in ARL8B–LC3 co-localization and impaired autophagosome maturation (Fig. 6P–S); and (3) robust suppression of viral particle secretion (Figure S5E–G). These findings were substantiated by cross-species validation in porcine (PK-15) and primate (Vero) cells, which consistently demonstrated ARL8B’s central role in coordinating lipid metabolism and vesicular transport across diverse single-stranded RNA viruses, including FMDV, VSV, poliovirus, and EV71. The evolutionary conservation of this mechanism highlights the universal dependence of single-stranded RNA viruses on ARL8B-mediated metabolic hijacking, particularly through regulation of Plin2-associated lipophagy and secretion pathways. The identification of HY-W051988 and HY-Q31266 represents the first pharmacological validation of ARL8B as a druggable target, offering a novel therapeutic strategy to disrupt viral exploitation of host metabolic networks through selective inhibition of this critical host factor. These compounds provide valuable chemical tools for studying ARL8B biology and establish proof-of-concept for host-directed development of broad-spectrum antivirals targeting conserved viral dependency factors.

### In vivo efficacy of the small-molecule inhibitor HY-W051988 in a mouse model of EV71 infection

In vivo experiments in this study employed EV71 instead of FMDV primarily because FMDV lacks a mature and reliable mouse infection model, whereas EV71 infection in suckling mice serves as a classic model for studying picornavirus replication and antiviral strategies in vivo. Given the high conservation between EV71 and FMDV in their replication mechanisms—particularly in the hijacking of host membrane lipid metabolism—this model effectively serves to evaluate the efficacy of antiviral strategies targeting the host factor ARL8B in vivo. To assess the susceptibility of C57BL/6J mice to EV71, 8-week-old mice were intranasally inoculated with varying doses of the EV71 wild-type strain. Mice were monitored daily for clinical symptoms, body weight changes, and post-infection survival. Under high-dose infection (3 × 10^5^ PFU), mice exhibited significant weight loss and substantially elevated viral loads in liver and kidney tissues (Figure S6A–B). Consequently, this high-dose inoculum (3 × 10^5^ PFU) was used in subsequent experiments. Given the broad-spectrum antiviral activity of small-molecule inhibitors, we examined the in vivo protective efficacy of HY-W051988. Eight-week-old C57BL/6J mice received 100 µL of HY-W051988 (10 mg/L in 90% corn oil) intraperitoneally. Measurements of body weight and survival confirmed that high concentrations of HY-W051988 had no adverse effects (Figure S6C).

**Fig. 7:**
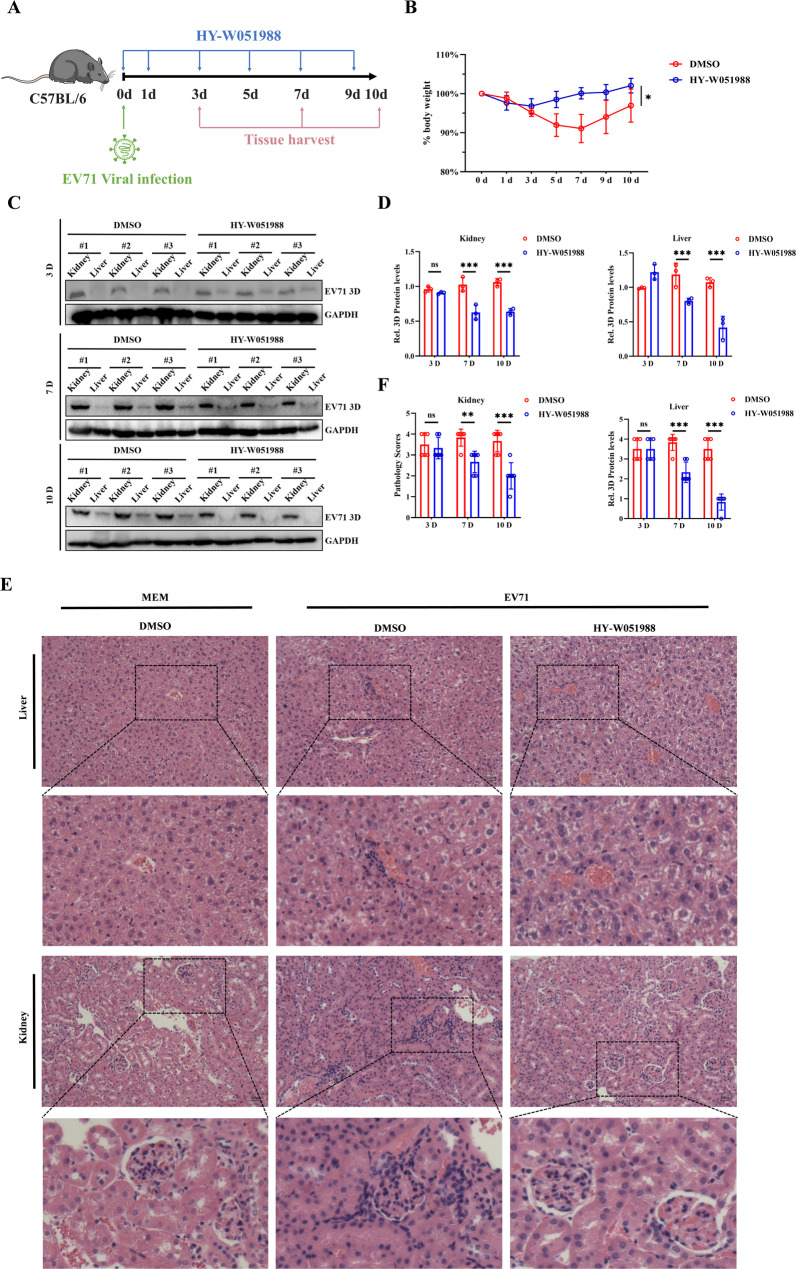
In vivo efficacy of the small-molecule inhibitor HY-W051988 in a mouse model of EV71 infection. (**A**) Experimental schematic of EV71 infection and HY-W051988 dosing. Eight-week-old C57BL/6J mice were anesthetized and intranasally inoculated with wild-type (WT) EV71. HY-W051988 was administered intraperitoneally at designated time points. (**B**) Body weight changes were monitored and evaluated in infected mice (*n* = 3). Mice were euthanized at the experimental endpoint for tissue collection. (**C–D**) Viral loads in liver and kidney tissues were detected by Western blotting (**C**) and quantified (**D**). (**E**) Hematoxylin and eosin staining of liver and kidney tissues from C57BL/6J mice after EV71 infection. Magnified regions highlight tissue damage. (**F**) Histopathological scoring of liver and kidney tissue injury. Data information: Scale bars, 30 μm. Values represent mean ± SD from three independent experiments. Statistical analysis was performed using t-tests (*n* = 3). *P < 0.05; n.s., not significant

To evaluate antiviral efficacy, C57BL/6J mice were intranasally inoculated with EV71 and then treated intraperitoneally with HY-W051988 (10 mg/kg) at 0, 1, 3, 5, 7, and 9 days post-infection, as well as the experimental endpoint (Fig. 7A). Liver and kidney tissues were collected at 3 and 7 days post-infection, along with the endpoint, for viral load determination. Viral loads were quantified at the endpoint, defined as death, decline in body weight to 80% of baseline, or completion of the experimental period. Mice treated with HY-W051988 exhibited transient weight loss at 3 days post-infection but recovered more rapidly than control animals (Fig. 7B). Compared with untreated controls, HY-W051988 significantly reduced viral loads in both liver and kidney tissues (Fig. 7C–D). Histopathological analysis revealed that EV71 infection induced severe interstitial hepatitis and nephritis in control mice, characterized by extensive inflammatory cell infiltration in the liver and kidneys, portal tract inflammation, renal capsule adhesion, and widespread perivascular inflammation. Mice treated with HY-W051988 displayed only mild pathological changes (Fig. 7E). These findings, consistent with molecular analyses, demonstrate that HY-W051988 treatment limits weight loss, viral replication, and organ injury in EV71-infected mice. HY-W051988 constitutes a robust antiviral compound with therapeutic potential against EV71.

## Discussion

To our knowledge, the present study is the first to show that FMDV activates the lysosome-dependent LD degradation pathway [41] by hijacking the host small GTPase ARL8B and regulating the lysosome–LD interaction network through a GTP-dependent conformational transition (Figs. 1 and 2), yielding essential lipids for viral replication (Fig. 3). Our findings establish ARL8B as a critical mediator of lysosome-dependent LD degradation. During FMDV infection, ARL8B promotes lipid turnover, resulting in accelerated LD formation (Fig. 2). Although FMDV infection did not significantly alter ARL8B expression levels, it induced ARL8B spatial aggregation through nonstructural protein 2 C (Figs. 1, S1), suggesting that viruses hijack lipid metabolism by remodeling the subcellular localization of host membrane transport proteins. Importantly, the GTP/GDP binding state of ARL8B (T34N/Q75L mutants) directly determines its capacity to anchor lysosomes or LDs [29] (Fig. 2). Disruption of this dynamic localization blocks LD degradation and inhibits viral replication (Fig. 2), thereby elucidating the molecular basis of ARL8B’s role as an “LD degradation switch” at the functional domain level.

Unlike porcine reproductive and respiratory syndrome virus, which upregulates Rab18-mediated autophagy of conventional LDs [42], or SARS-CoV-2, which activates syntaxin 18–dependent lipophagy through its M protein [43], FMDV uniquely exploits ARL8B-regulated direct lysosome–LD interactions (Fig. 2). This mechanism bypasses autophagosome encapsulation and enables rapid lipid release. The present study is the first to demonstrate that FMDV infection activates lysosome-mediated lipophagy in host cells via ARL8B, as revealed by multimodal imaging analyses (LAMP1/Bodipy/LC3 co-localization; Fig. 3). In summary, our findings establish ARL8B as a critical metabolic regulator during FMDV infection that activates lysosome-dependent lipophagy. This pathway releases host lipids that are subsequently hijacked by the virus and redirected to replication sites, where they provide essential lipids for replication complex membrane remodeling. Unlike conventional autophagy-dependent lipophagy, such as rapamycin-induced global degradation of LDs [44, 45], FMDV-triggered LD degradation displayed strong spatial specificity. Autophagic lysosomes preferentially aggregated in the perinuclear region, where they formed close membrane contacts with the endoplasmic reticulum and Golgi intermediates (Figs. 4A, S3A). This localization closely paralleled the spatial and temporal distribution of FMDV replication complexes, suggesting that the virus achieves “targeted delivery of lipid resources” by hijacking the perinuclear membrane system. Although the autophagy inhibitor CQ blocked conventional lipophagy (indicated by LC3 + autophagosome accumulation; Fig. 3K) and reduced viral replication (Fig. 4E), these findings indicate that FMDV has evolved an ARL8B-dependent “LD degradation pathway.” This mechanism helps explain the persistent paradox whereby global activation of autophagy (e.g., via starvation) can suppress infection through antiviral signaling [46]; in contrast, FMDV manipulates compartmentalized autophagy in the perinuclear region to create a “pro-viral autophagic microenvironment.” Single-cell imaging of autophagy subtype dynamics further supports this model and may elucidate the molecular basis of the “double-edged sword of autophagy” phenomenon. The physical interaction of ARL8B with LC3 (Fig. 4M–N) and its enrichment at lysosome–LD contact sites (Fig. 3) likely mediate targeted secretion of lysosomal enzymes, such as acid lipase, through the HOPS complex. This process directly hydrolyzes LD triglycerides to release free fatty acids that comprise substrates for viral membrane synthesis. Future structural resolution of the ARL8B–LC3–2C ternary complex will yield detailed mechanistic insights regarding this viral lipid acquisition strategy. An understanding of the ARL8B–LC3 interface provides a molecular framework for explaining how viral pathogens exploit host membrane trafficking systems via targeted manipulation of key protein interaction nodes.

**Fig. 8:**
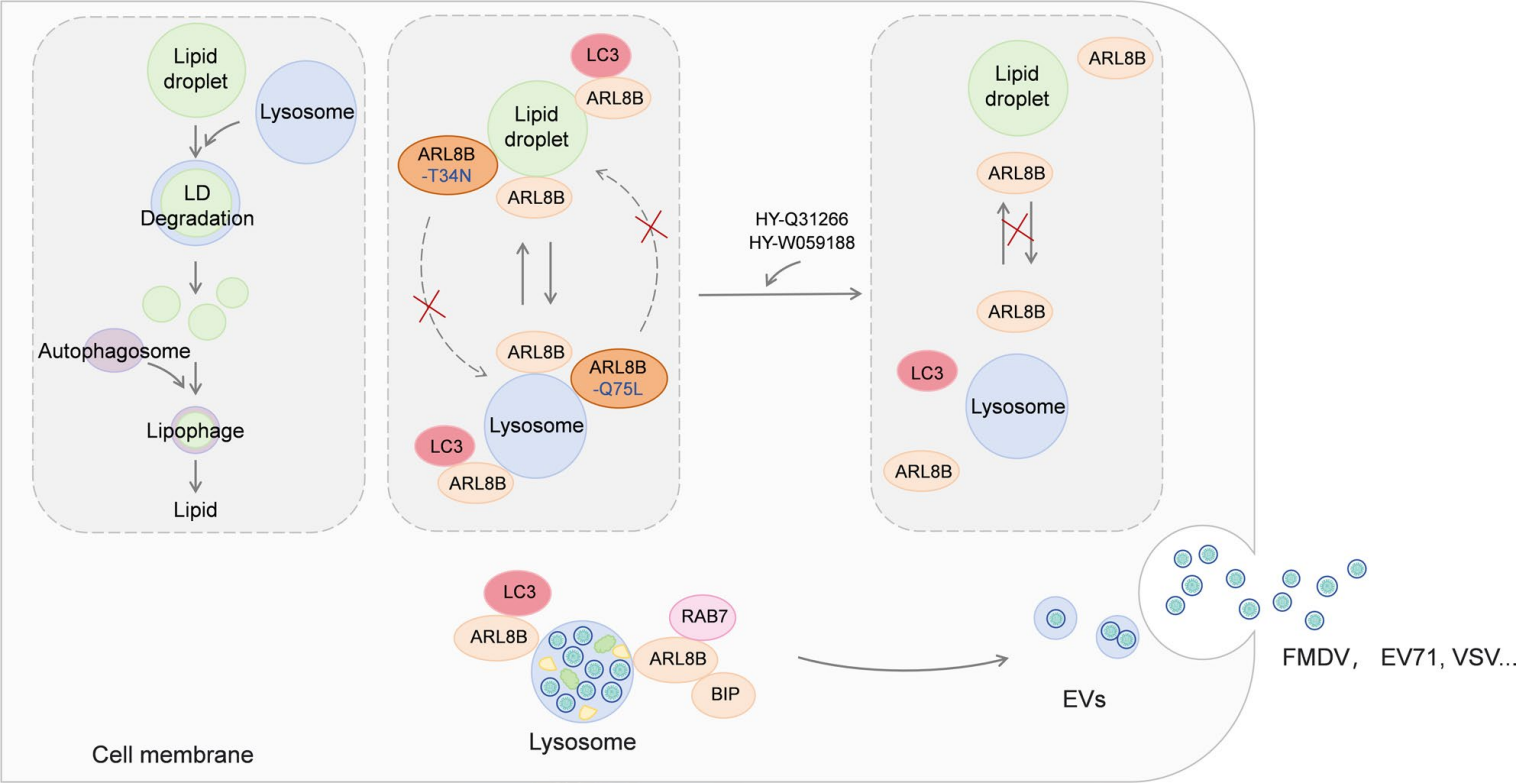
Host factor ARL8B regulates the replication and transmission of multiple RNA viruses through dual functions: initiating lipophagy and mediating lysosome–exosome release

Viruses can increase EV production or manipulate EVs to facilitate their spread, as shown for hepatitis A virus [47], hepatitis E virus [48, 49], EV71 [50], and bluetongue virus [51, 52]. These “quasi-enveloped” viruses evade immune recognition and broaden their cell tropism while egressing in a non-cytolytic manner. EVs are secreted through intraluminal vesicles within MVBs. This biosynthetic pathway begins with endosome maturation into MVBs, where cargo-recruitment factors induce inward budding of the endosomal membrane and intraluminal vesicle formation. Cargoes may include proteins, nucleic acids, metabolites, and lipids [53, 54]. In this study, exosomal properties of EVs were verified by marker profiling (CD63+/CD81+/TSG101+/calnexin−) and TEM, consistent with MISEV2018 standards (Fig. 5E). Our results confirm the hypothesis that FMDV achieves “covert transmission” by hijacking the exosomal secretion system. Importantly, specific enrichment of viral nonstructural proteins 2C and 3D in exosomes suggests that packaging occurs through an ESCRT-III complex-mediated intraluminal vesicle sorting mechanism. This process resembles coronavirus M-protein–regulated vesicular transmission, such as ESCRT-dependent sorting of the SARS-CoV-2 S protein [55], and reflects convergent evolutionary adaptation. We found that FMDV hijacks the host EV secretion system through a dual synergistic mechanism: classical Golgi-dependent secretion pathway activation (Figure S4J) and ARL8B-regulated lysosome–EV nonclassical transport pathway exploitation (Fig. 5). In the latter pathway, ARL8B establishes endoplasmic reticulum–lysosome membrane contact sites (ERLCS) through BIP–ARL8B interactions (Figure S4B), enabling virus-directed capsid transport. This regulatory mechanism confers exceptionally high secretion efficiency. ARL8B knockdown caused a substantial reduction in viral EV load (Fig. 5F), a decrease far greater than that observed upon inhibition of the classical secretory pathway (40% reduction with CID treatment; Figure S4C), indicating that the lysosome–EV pathway serves as a primary driver of viral transmission. This strategy contrasts with the approach of human immunodeficiency virus, which utilizes a single quasi-enveloped pathway via Rab protein–mediated MVB bypass, or with the mechanisms of hepatitis A virus/hepatitis E virus [56]; it highlights the unique membrane transport plasticity evolved by non-enveloped viruses to overcome host restrictions. Importantly, we identified Annexin V as a novel specific receptor for FMDV-associated EVs. In vitro treatment with Annexin V antibody reduced EV-mediated infection efficiency by up to 90% (Fig. 5E), providing a molecular basis for the development of EV-targeted antiviral blockers. Additionally, structured illumination microscopy dynamic imaging revealed that ARL8B modulates EV release through localized membrane curvature, facilitating viral outgrowth (Fig. 5J). Although the present study established a central role for ARL8B in this process, the molecular details of its regulation of ERLCS formation [55] and BIP transport warrant further exploration. Based on these findings, we propose a model for ARL8B-mediated regulation of picornavirus replication and transmission (Fig. 8). Host factor ARL8B occupies a central position in the life cycle of multiple single-stranded RNA viruses by coordinating two essential cellular processes: initiation of lipophagy and facilitation of lysosome–exosome release. Through its GTPase activity and interactions with autophagy-related proteins such as LC3, ARL8B promotes LD degradation to supply critical lipid substrates for viral replication. Concurrently, it supports extracellular release of viral particles through exosomal pathways via lysosome-dependent secretion. This dual mechanism enables efficient viral replication and intercellular transmission, identifying ARL8B as a key host factor used to enhance the propagation and spread of single-stranded RNA viruses (Fig. 1).

This study validated ARL8B as an effective antiviral target in foot-and-mouth disease virus (FMDV) and enterovirus 71 (EV71). Both viruses belong to the Picornaviridae family, exhibiting highly conserved genomic replication mechanisms. Therefore, our findings provide robust proof-of-concept for developing novel antiviral strategies within this viral family. While targeting the host factor ARL8B holds conceptual potential for countering other viruses that rely on similar membrane remodeling mechanisms (e.g., coronaviruses, flaviviruses), this “broad-spectrum” hypothesis requires future direct experimental validation across different viral families. Conventional anti-RNA virus drug development has mainly focused on viral targets, such as the chikungunya virus nsP2 protease (with inhibitor KJ-98 A identified through computer-based virtual screening) [57–60] or SARS-CoV-2 NSP14 (with TDI-015051 tested in lung organoids) [61]. Although these drugs exhibit strong antiviral efficacy, their clinical application faces two major challenges. First, the high mutation rates of viral RNA polymerases lead to rapid emergence of drug resistance, resulting in target escape (e.g., the hepatitis C virus NS5B inhibitor sofosbuvir exhibits a resistance mutation rate of approximately 10%) [62]. Second, there are limitations in broad-spectrum coverage, given that protein-targeting strategies often fail across viral species (e.g., coronavirus 3CLpro inhibitors are ineffective against enteroviruses) [63]. Here, we developed small-molecule inhibitors (HY-Q31266 and HY-W051988) targeting the host regulatory hub ARL8B, which offer distinct advantages. First, both compounds exhibit broad-spectrum activity against RNA viruses from the Picornaviridae and Rhabdoviridae families in vitro (Fig. 6). Second, because ARL8B is a host protein with an extremely low mutation rate, and the inhibitors target its functional domain rather than a classical binding pocket, the risk of viral escape through single-point mutations is reduced. These compounds disrupt the viral replication microenvironment by simultaneously blocking lipophagy (Fig. 6H–K), thereby cutting off the supply of essential lipids required for replication. Additionally, they inhibit viral particle secretion (Figure S5E–G), producing a synergistic, multi-pathway antiviral effect. This study represents the first application of molecular dynamics simulations combined with structure-guided screening to identify ARL8B-targeting small molecules, demonstrating broad antiviral efficacy and highlighting the promise of host-based strategies. Initial in vivo evidence from an EV71-infected mouse model shows that HY-W051988 reduces viral replication in multiple organs and alleviates pathology (Fig. 7). By disrupting essential host pathways hijacked by RNA viruses, ARL8B inhibitors provide broad-spectrum activity while circumventing the resistance issues common to conventional approaches. With advanced delivery technologies and rational drug combinations, such agents hold potential for clinical translation, offering a strategic therapeutic reserve against emerging viral outbreaks.

## Supplementary Information

Below is the link to the electronic supplementary material.

Supplementary Material 1

Supplementary Material 2

Supplementary Material 3

Supplementary Material 4

Supplementary Material 5

Supplementary Material 6

Supplementary Material 7

## Data Availability

Data that support the findings of this study are available from the corresponding author upon reasonable request.
