# Supplementary figures and images for: ARF-like GTPase 8B orchestrates lipophagy and exocytosis to drive single-stranded RNA virus replication
Source: J Transl Med. 2026 Jun 13;24:880. doi: 10.1186/s12967-026-08417-2 (PMC13355346; doi:10.1186/s12967-026-08417-2)

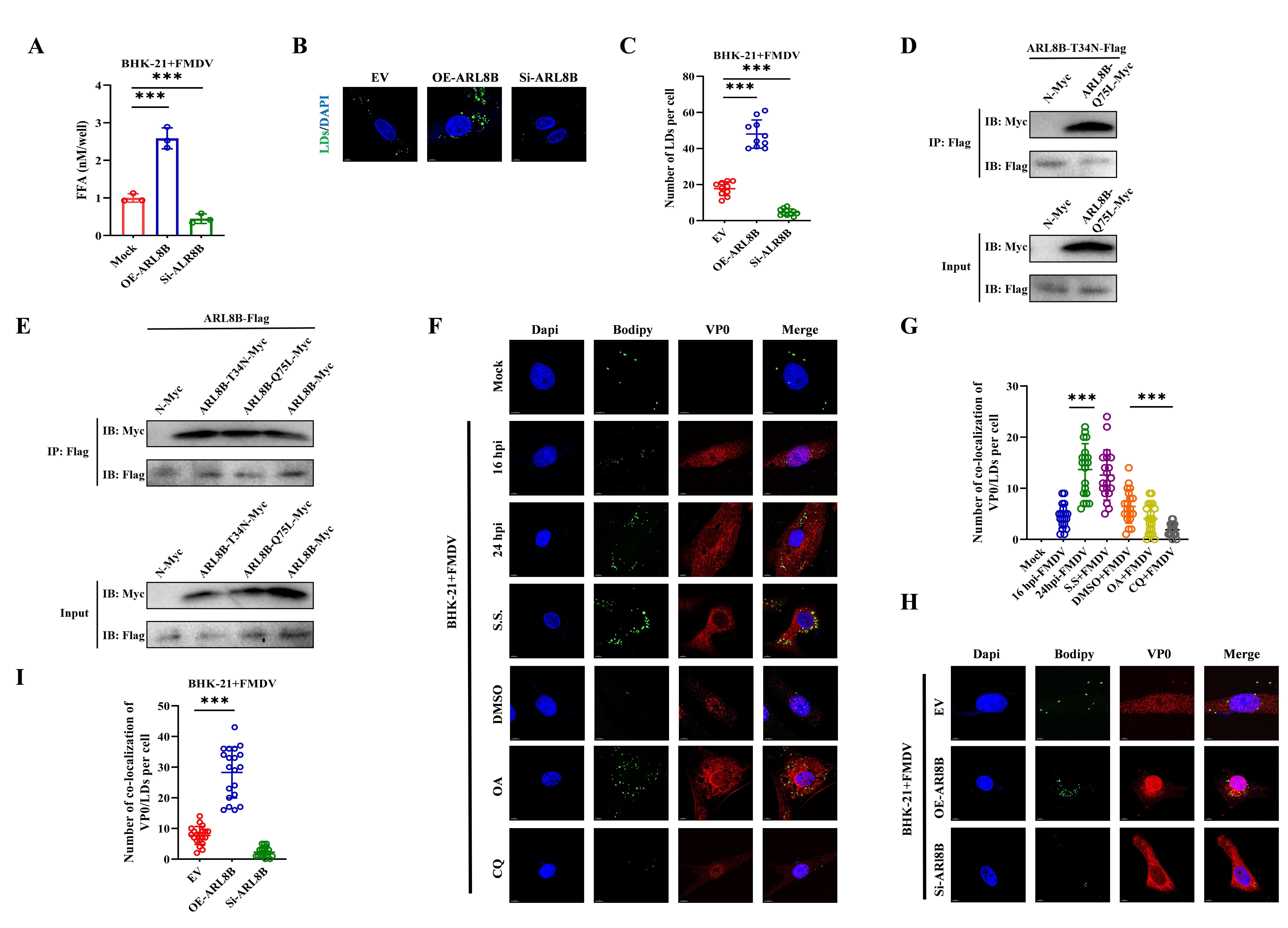

Supplement: Supplementary file 1 — Supplementary Material 1 [file 12967_2026_8417_MOESM1_ESM.tif]

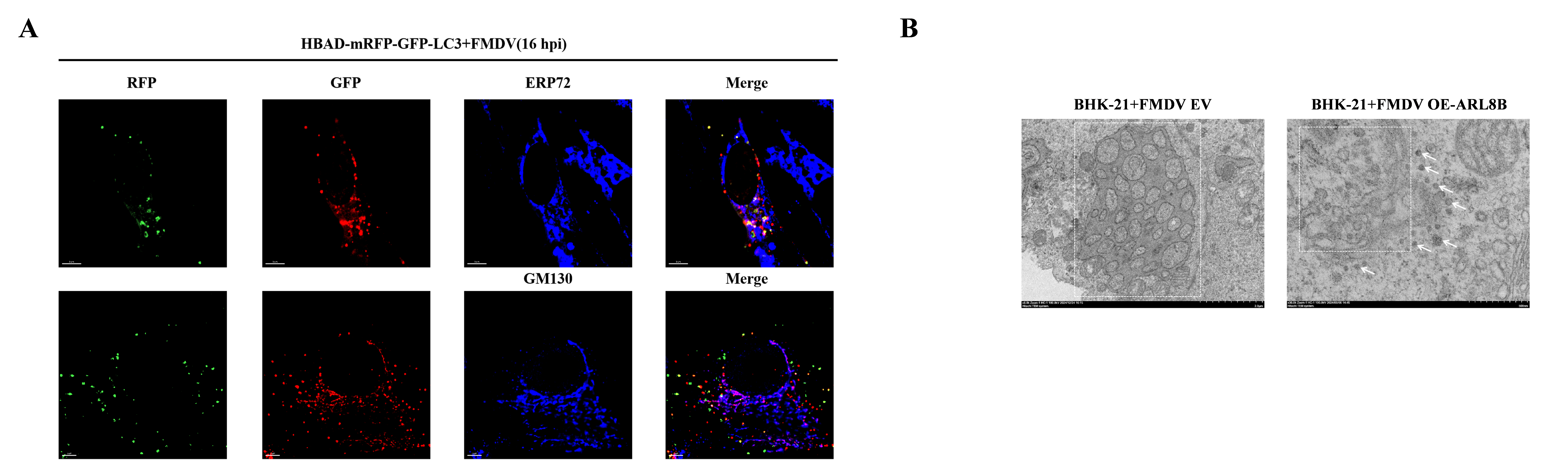

Supplement: Supplementary file 2 — Supplementary Material 2 [file 12967_2026_8417_MOESM2_ESM.tif]

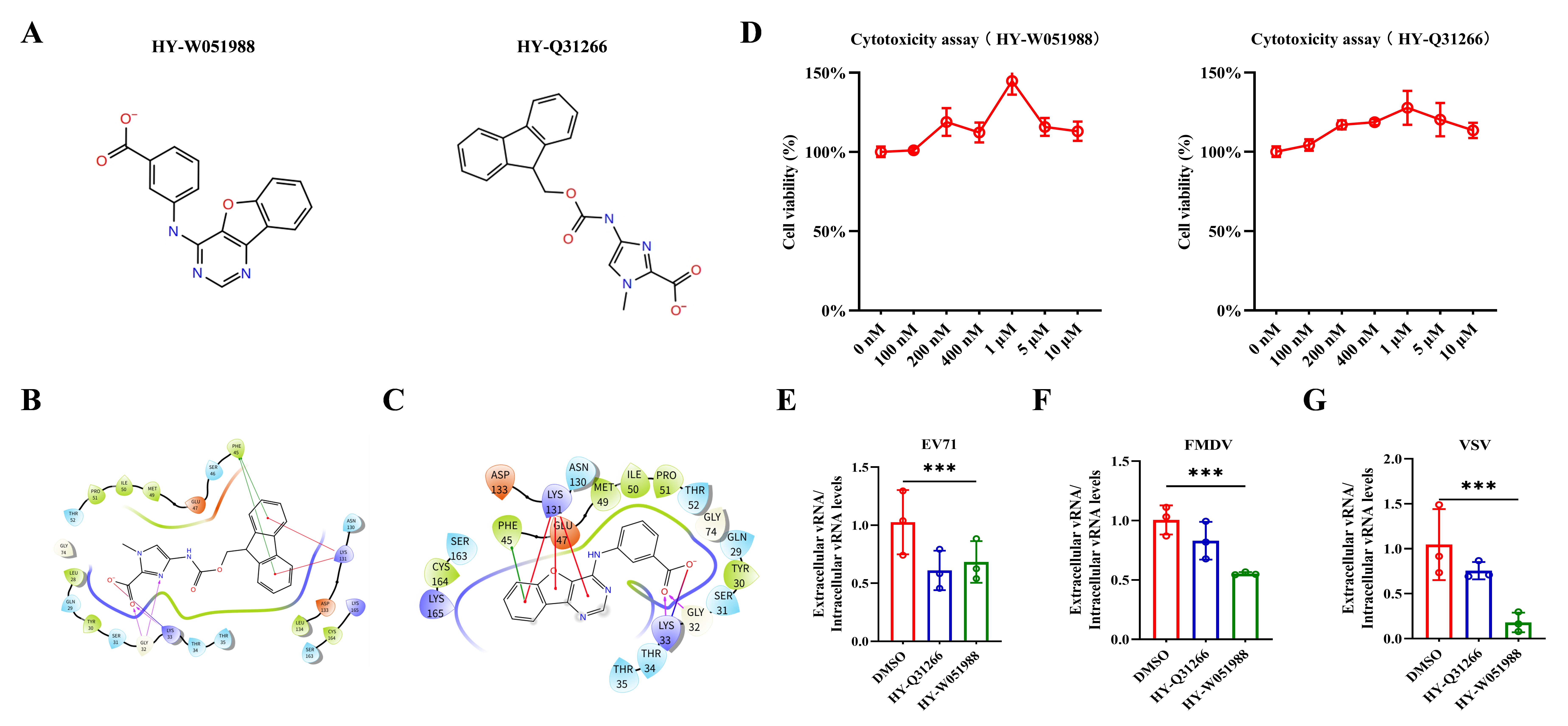

Supplement: Supplementary file 4 — Supplementary Material 4 [file 12967_2026_8417_MOESM4_ESM.tif]

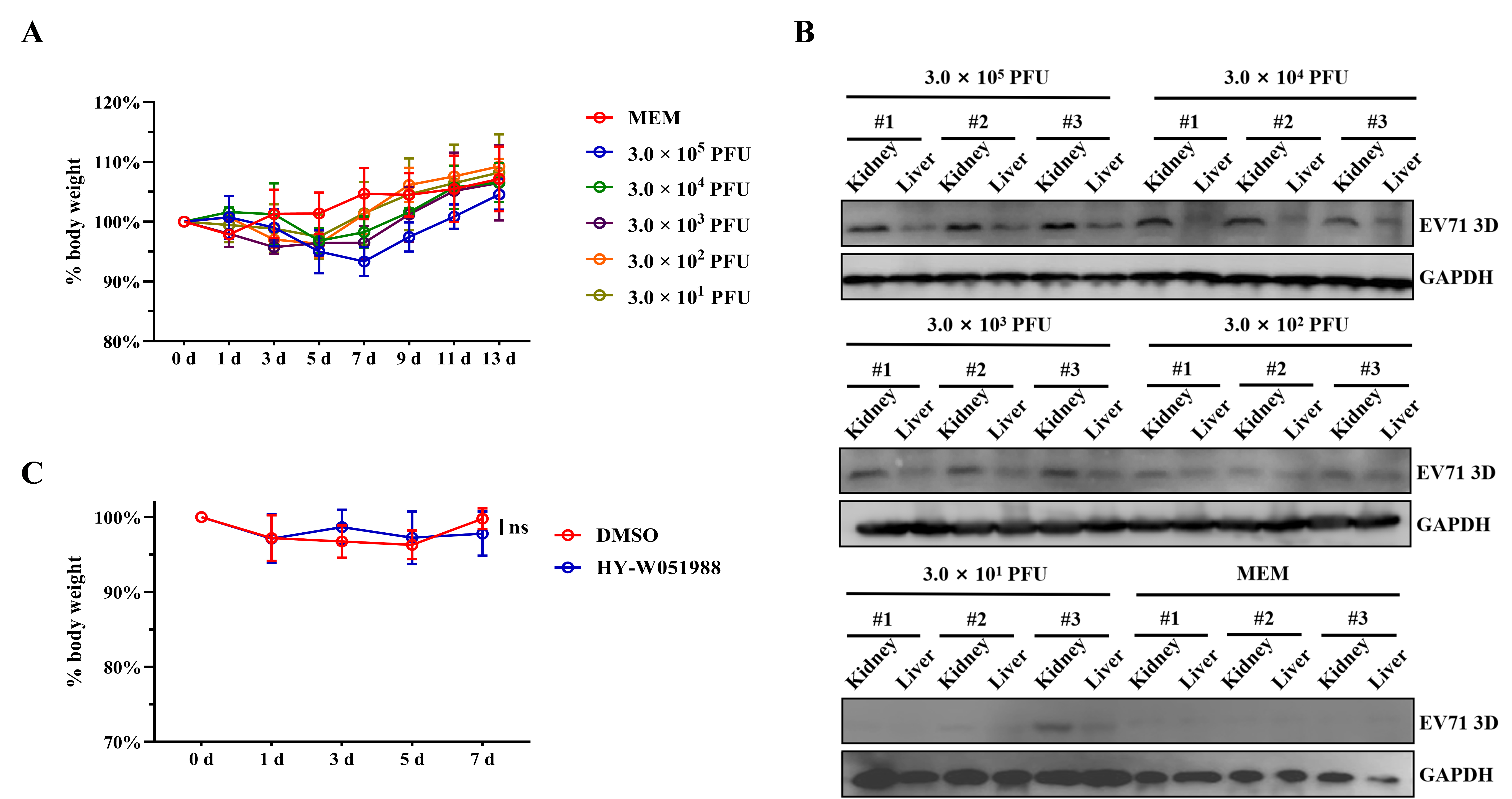

Supplement: Supplementary file 5 — Supplementary Material 5 [file 12967_2026_8417_MOESM5_ESM.tif]

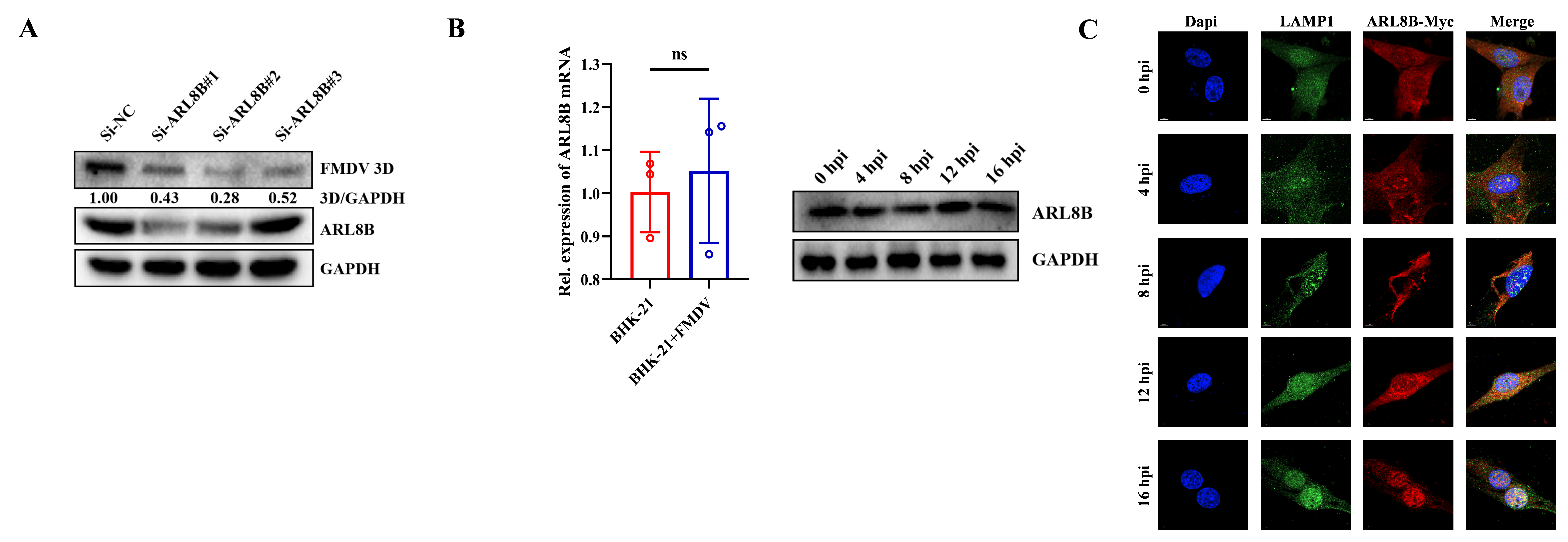

Supplement: Supplementary file 6 — Supplementary Material 6 [file 12967_2026_8417_MOESM6_ESM.tif]
