# Supplementary material for: ARF-like GTPase 8B orchestrates lipophagy and exocytosis to drive single-stranded RNA virus replication
Source: J Transl Med. 2026 Jun 13;24:880. doi: 10.1186/s12967-026-08417-2 (PMC13355346; doi:10.1186/s12967-026-08417-2)
Supplement: Supplementary file 7 — Supplementary Material 7 [file 12967_2026_8417_MOESM7_ESM.doc]

**ARF-like GTPase 8B** **Orchestrates Lipophagy and Exocytosis to Drive Single-stranded RNA Virus Replication**

Bonan Lv^a, b^, Xingran Wang^a, b^, Ying Zhou^a, b^, Zihan Su^a, b^, Yidan Sun^a, b^, Li Zhou^c^, Yang Lu^b^, Zishu Pan^a^, Xiao-Feng Tang^b^, Chao Shen^a, b, *^

^a^State Key Laboratory of Virology and Biosafety, College of Life Sciences, Wuhan University, Wuhan 430072, China

^b^Hubei Key Laboratory of Cell Homeostasis, College of Life Sciences, Wuhan University, Wuhan 430072, China

^c^ABSL-III Laboratory at Center for Animal Experiment, Wuhan University School of Medicine, Wuhan 430071, China

^*^Corresponding authors: Chao Shen (shenchao@whu.edu.cn)

**Supplemental information**


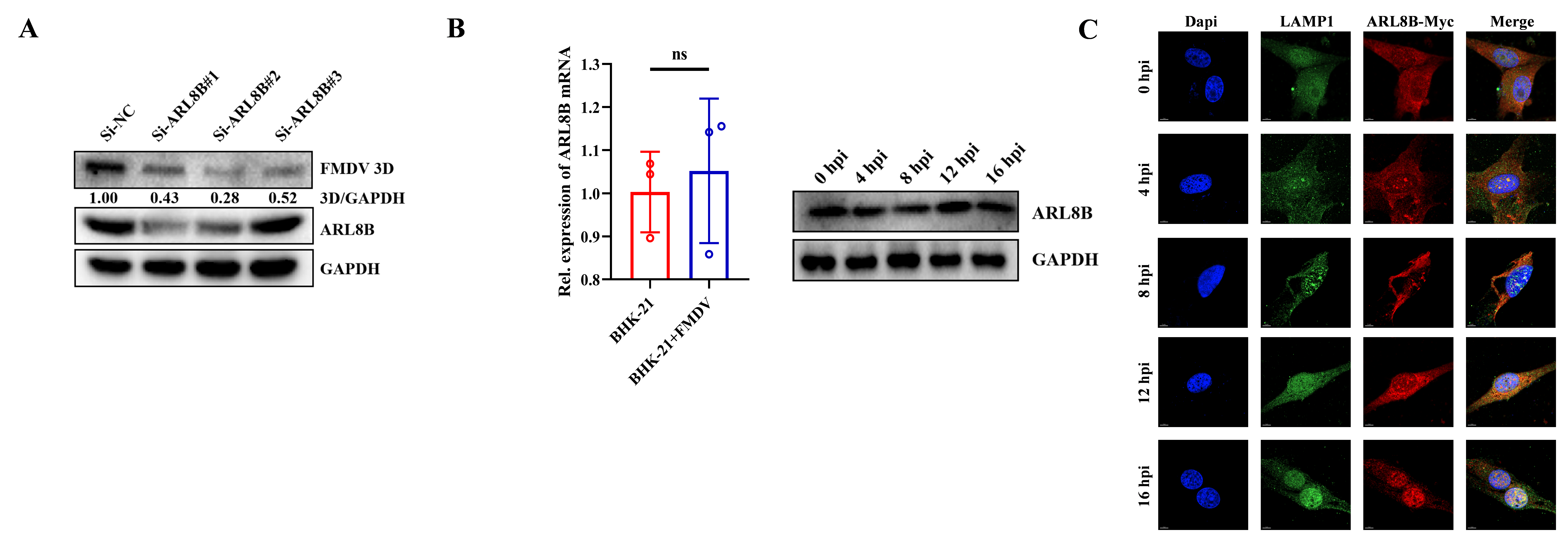


**Figure S1**

(A) Cells were transfected with the indicated siRNAs for 24 h and infected with FMDV (MOI = 1) for 16 h. Cell lysates were analyzed by Western blotting. (B) BHK-21 cell cDNA was extracted with or without FMDV infection (MOI = 1) for 16 h; ARL8B RNA and protein levels were determined by RT-qPCR and immunoblotting. (C) BHK-21 cells were infected with FMDV (MOI = 1) for 16 h, and samples were collected at the indicated time points for Western blot analysis of cell lysates. Data information: Scale bars, 5 μm. Values represent mean ± SD from three independent experiments. Statistical analysis was performed using t-tests (n = 3). *P < 0.05; n.s., not significant.

**
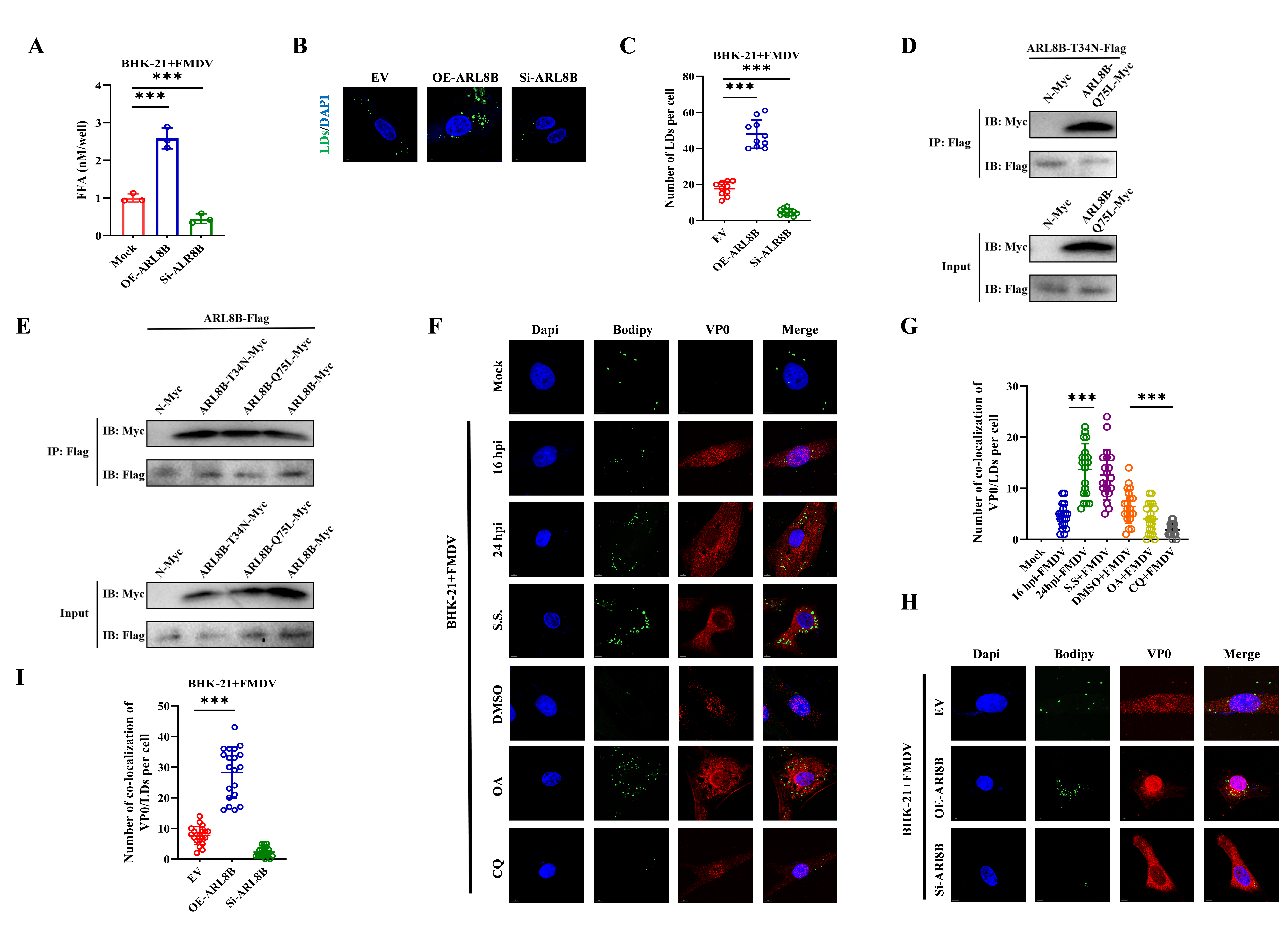
**

**Figure S2**

1. BHK-21 cells were transfected with the indicated plasmids for 24 h and infected with FMDV (MOI = 1, 16 h). Free fatty acid concentrations were measured in cell lysates. (B–C) BHK-21 cells were transfected with the indicated plasmids for 24 h and infected with FMDV (MOI = 1, 16 h). Cells were fixed and stained with Bodipy 493/503 (green) to label LDs and DAPI (blue) for nuclei, then imaged by confocal microscopy. The number of LDs per cell was quantified from 10 cells across three independent experiments. (D) ARL8B self-interaction was assessed by co-immunoprecipitation. (E) Interactions among ARL8B mutants were analyzed by co-immunoprecipitation. (F, H) BHK-21 cells were treated under the indicated conditions, then fixed and stained with anti-FMDV VP0 antibody (red) for viral granules, Bodipy (green) for LDs, and DAPI (blue) for nuclei. Images were obtained by confocal microscopy. (G, I) Quantification of VP0/LD co-localization events per cell from 20 cells across three independent experiments, corresponding to (F, H). Data information: Scale bars, 5 μm. Values represent mean ± SD from three independent experiments. Statistical analysis was performed using t-tests (n = 3). *P < 0.05; n.s., not significant.

**
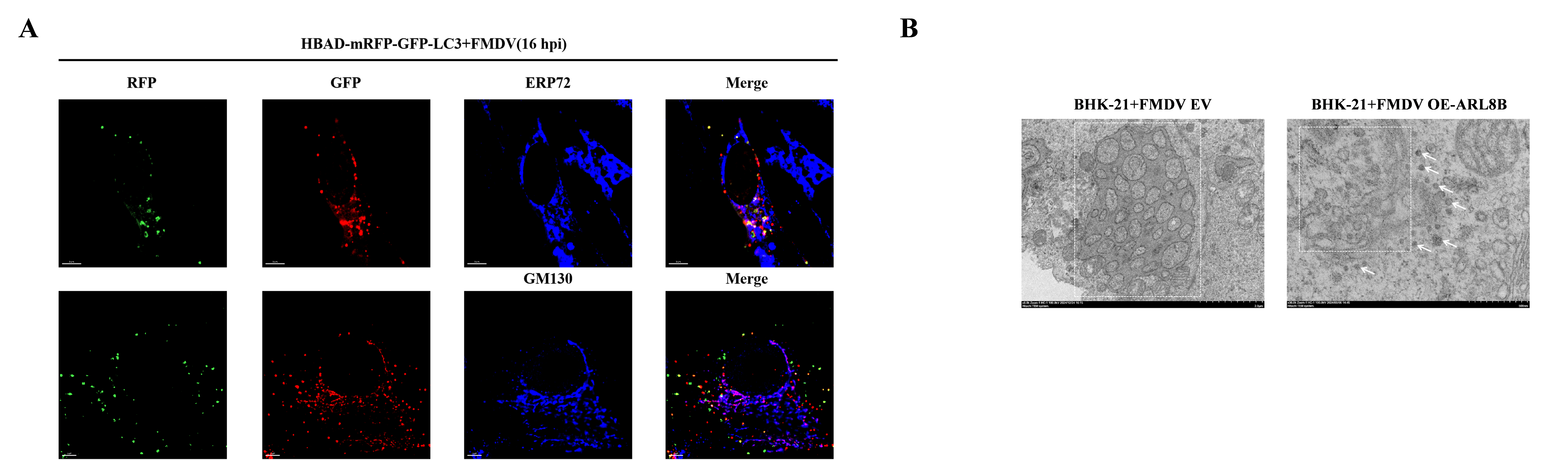
**

**Figure S3**

(A) BHK-21 cells expressing mRFP-GFP-LC3 were treated for 48 h under the indicated conditions. The endoplasmic reticulum was stained with ERP72; the Golgi apparatus was stained with GM130 (blue). Cells were fixed and imaged using confocal microscopy. (B) Transmission electron micrographs of BHK-21 cells infected with FMDV (MOI = 1, 16 h). White dashed box highlights the endoplasmic reticulum, and white arrowheads indicate autophagic vesicles. Data information: Scale bars, 5 μm. Values represent mean ± SD from three independent experiments. Statistical analysis was performed using t-tests (n = 3). *P < 0.05; n.s., not significant.

**Figure S4**

(A) Negative-staining transmission electron micrographs of FMDV viral particles obtained from conventionally lysed cells vs. FMDV particles encapsulated within cryo-crushed EVs. (B) BHK-21 cells infected with FMDV (MOI = 1, 16 h) were fixed and then permeabilized or not permeabilized. Cells were stained with ARL8B antibody (green) and BIP antibody (red). (C) BHK-21 cells infected with FMDV (MOI = 1, 16 h) were treated with CID; cell lysates were analyzed by Western blotting. (D) BHK-21 cells infected with FMDV (MOI = 1, 16 h) were treated with BFA, and cell lysates were analyzed by Western blotting. (E–G) Determination of IC_50_ values for BFA, CID, and CQ. (H) Following addition of BFA or CID to BHK-21 cells or Si-ARL8B cells, RNA was extracted from EVs 16 h after FMDV infection (MOI = 1); FMDV vRNA content was quantified by RT-qPCR. (I) BHK-21 cells infected with FMDV (MOI = 1, 16 h) and treated with BFA were assayed for Gaussia luciferase activity. (J) BHK-21 or KO-ARL8B cells were infected with FMDV (MOI = 1, 16 h), treated with BFA, and analyzed to determine extracellular vRNA proportions. (K) Scanning electron microscopy of control cells (Si-NC), FMDV-infected cells (Si-NC + FMDV), and ARL8B-knockdown FMDV-infected cells (Si-ARL8B + FMDV). Quantitative analyses of exosome diameter (L), number (M), and diameter distribution by distance from nucleus (N) are shown at right. n = 20. (O) Vero cells expressing mRFP-GFP-LC3 were treated under the indicated conditions for 48 h, then fixed and imaged by confocal microscopy. (P) Quantification of RFP and GFP co-localization events per cell, based on 20 cells from three independent experiments. (Q) Vero cells expressing mRFP-GFP-Plin2 were treated under the indicated conditions for 48 h, then fixed and imaged by confocal microscopy. (R) Quantification of RFP and GFP co-localization events per cell, based on 20 cells from three independent experiments. (S–T) EV71 and VSV extracellular vRNA/intracellular vRNA ratios were determined by RT-qPCR after BFA and CID treatment. (U–V) Cells transfected with Si-NC or Si-ARL8B for 24 h and infected with EV71 or VSV for 24 h were analyzed by RT-qPCR to determine extracellular vRNA/intracellular vRNA ratios. (W–X) BHK-21 cells infected with EV71 or VSV (MOI = 1, 24 h) and treated with CID or BFA were analyzed to determine Gaussia luciferase activity. Data information: Scale bars, 200 nm (A), 2 µm (K), or 5 μm (others). Values represent mean ± SD from three independent experiments. Statistical analysis was performed using t-tests (n = 3). *P < 0.05; n.s., not significant.


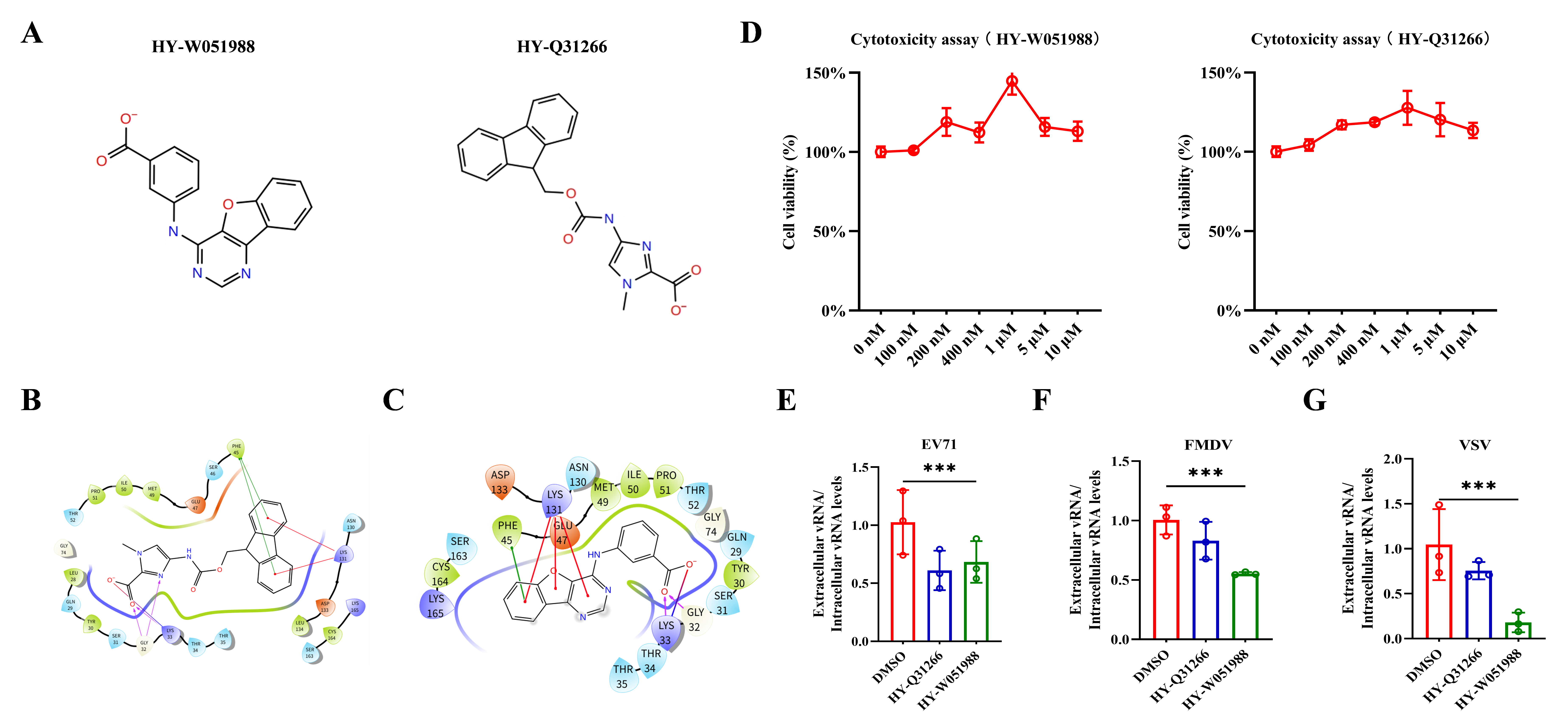


**Figure S5**

(A) Structural representations of HY-W051988 and HY-Q31266. (B) ARL8B Protein Binding to HY-W051988: 2D Structure Visualization. (C) ARL8B Protein Binding to HY-Q31266: 2D Structure Visualization. (D) Determination of infectious dose 50% (IC_50_) values for HY-W051988 and HY-Q31266. (E–G) Quantification of extracellular and intracellular vRNA levels of EV71, FMDV, and VSV by RT-qPCR after treatment with HY-Q31266 and HY-W051988. Data information: Values represent mean ± SD from three independent experiments. Statistical analysis was performed using t-tests (n = 3). *P < 0.05; n.s., not significant.

**
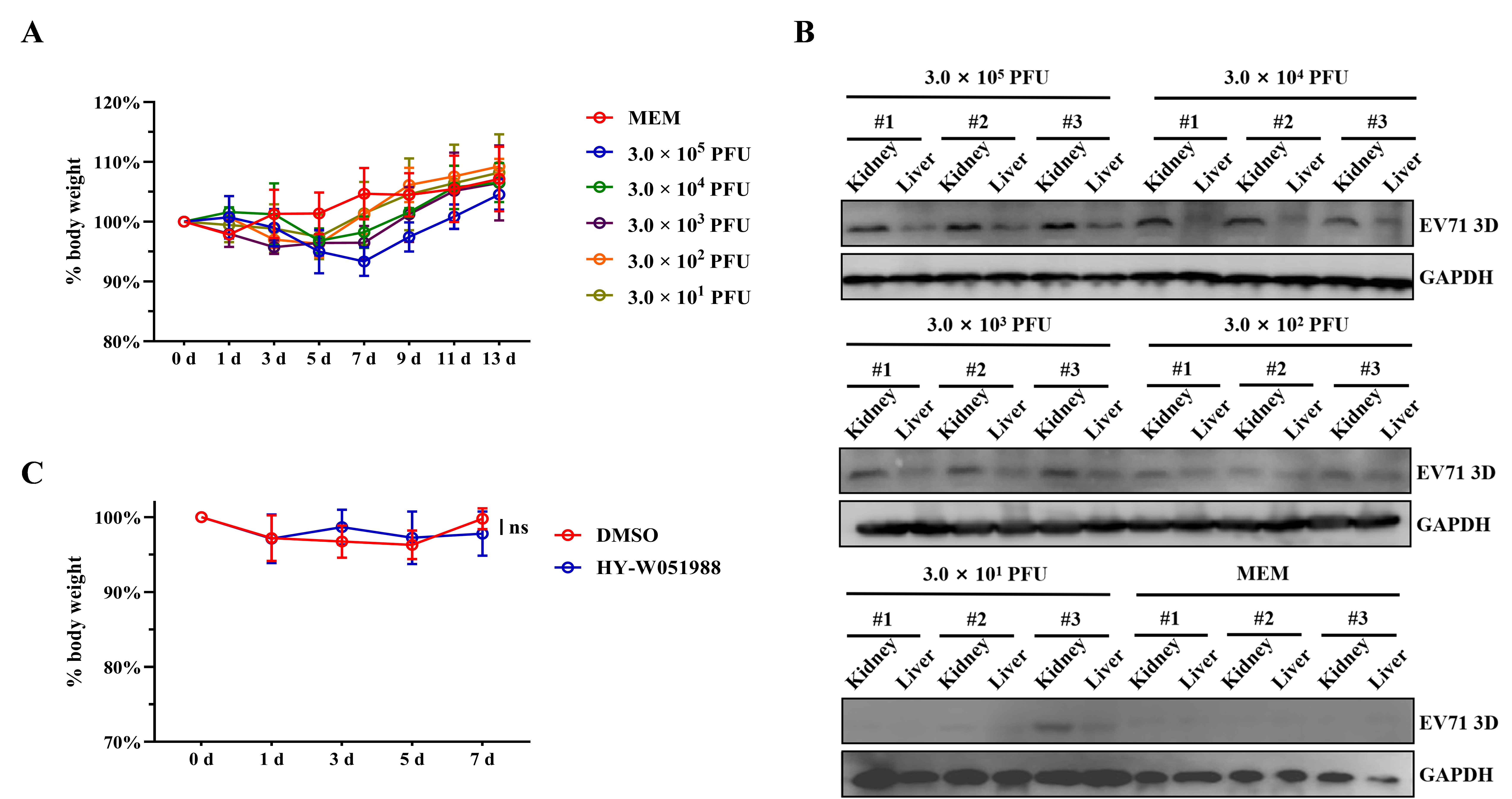
**

**Figure S6**

Low-dose EV71 infection in C57BL/6J mice caused changes in body weight (A) and viral load (B). (C) Safety evaluation of the small-molecule inhibitor HY-W051988 in C57BL/6J mice. Data information: Values represent mean ± SD from three independent experiments. Statistical analysis was performed using t-tests (n = 3). *P < 0.05; n.s., not significant.
